# Demystifying Quality Metrics and Unveiling the True Measure of Quality of Care in Nursing Homes: Mixed Effects Analysis

**DOI:** 10.2196/72770

**Published:** 2026-01-29

**Authors:** Suhas Bharadwaj, Haneen Ali

**Affiliations:** 1 Department of Industrial and Systems Engineering, College of Engineering, Auburn University, Auburn, AL, United States; 2 Department of Industrial and Systems Engineering, College of Science and Engineering, University of Minnesota, Twin Cities, Minneapolis, MN, United States; 3 Mechanical and Industrial Engineering Department, Faculty of Engineering and Technology, Applied Science Private University, 21 Al Arab Street, Amman, 11937, Jordan, 00962-65609999

**Keywords:** 5-Star Quality Rating, nursing homes, performance measures, outcomes, COVID-19, nursing home industry, nursing home performance, nursing home quality, quality measures, SPO, structure, process, or outcome, Donabedian SPO framework

## Abstract

**Background:**

The 5-Star Quality Rating System for nursing homes plays a central role in evaluating quality of care, although it has both strengths and limitations. This system relies heavily on the Minimum Data Set and derives several quality measures (QMs) from it. In this study, we validated the effectiveness of the 5-Star Quality Rating System for nursing homes and its underlying QMs in estimating quality of care. We constructed a panel dataset of US nursing homes (n=15,416) active from May 2020 to June 2023, retrieving data from three major sources: (1) COVID-19 nursing home data, (2) Payroll-Based Journal data, and (3) nursing home QM snapshots. The outcome variables included (1) resident infection, (2) staff infection, or (3) resident and staff deaths. The predictor variables were the 5-Star Quality Rating System for nursing homes and its underlying QMs classified as structure, process, or outcome (SPO) QMs.

**Objective:**

This study aims to evaluate the effectiveness of nursing home QMs by regressing nursing home COVID-19 outcomes on nursing home QMs, classified using the Donabedian SPO framework. We hypothesized that nursing homes with better structural quality (eg, greater staff availability, better skill mix, and so on), better process quality (eg, lower restraint use and higher vaccination rates), and better outcome quality (eg, lower number of residents with pressure ulcers and a lower number of resident falls) experienced better COVID-19 performance in terms of resident and staff infections and deaths.

**Methods:**

To examine the association between the COVID-19 outcomes and SPO QMs, we imputed missing values in the dataset using random forest. Subsequently, we modeled the imputed dataset using hurdled zero-inflated negative binomial mixed effects models. The zero inflation model included factors influencing initial susceptibility to COVID-19 or factors influencing the possibility of death after COVID-19 had been contracted. The model estimates were conditioned on zero inflation and random effects.

**Results:**

Staffing measures (*P*<.001 for all variables in all models), health deficiency scores (*P*<.001 for all variables in at least 1 model), COVID-19 hospitalization rates (*P*<.001 for all variables in at least 2 models), and vaccinations (*P*<.001 for all variables in at least 2 models) exhibited meaningful relationships with the COVID-19 outcomes, while the 5-star components, Medicaid dependency, and ownership showed no clear relationships.

**Conclusions:**

Although widely used, the 5-Star Quality Rating System for nursing homes is an unreliable performance measure. Concerted efforts from lawmakers, policy makers, and lobbyists are needed to refine and enhance the measure, thereby ensuring its reliability and effectiveness.

## Introduction

In the United States, nursing homes are essential care providers for more than 1.3 million residents across 15,600 facilities [1,2]. As the third-largest sector in the health care industry, nursing homes employ more than 1.7 million dedicated staff members [2] who cater to the complex needs of the older adults, who are often frail and affected by multimorbidity [3-6]. With projections indicating consistent increases in the older adult population [7] and average life expectancy [8,9], the demand for nursing home care is expected to rise sharply [8,10,11]. This makes it crucial for nursing homes to optimize their resource allocation while providing excellent care. Embracing the concept of quality of care (QoC) is not just a strategic necessity for survival in a competitive market but also a moral obligation to ensure the well-being of older adults.

The United States recorded 649,611 excess deaths more than expected in the first year of the COVID-19 pandemic, a 23% increase over the previous year. The largest share of excess deaths occurred among older adults [12]. In the nursing home setting, older adults were disproportionately affected [2] because of a combination of individual factors, such as advanced age [13,14] and comorbidities [14,15], and organizational factors, such as understaffing, crowding [2], limited supply of personal protective equipment [16], and inadequate infection prevention and control readiness [2,17,18]. While the Centers for Disease Control and Prevention proposed rigorous COVID-19 control measures, including maintaining personal hygiene practices, mask use, self-isolation, mobility restrictions, and physical distancing [19,20], they could not compensate for the chronic noncompliance of infection prevention and control practices. A May 2020 United States Government Accountability Office report found that 82% of Centers for Medicare and Medicaid Services (CMS)–certified nursing homes had at least one infection-related deficiency between 2013 and 2017, with nearly half receiving citations over multiple years [17,21].

Aimed at deterring noncompliance and promoting accountability, CMS launched the Nursing Home Care (NHC) website in 2002 to improve transparency by expanding public access to quality information [22]. However, despite CMS’s efforts, this website is not well known and is considered difficult to comprehend. When consulting NHC, consumers are unable to discern meaningful differences between nursing homes to make informed decisions about their well-being and that of their loved ones. To address this problem, the CMS introduced the 5-Star Quality Rating System for nursing homes in 2008, summarizing the detailed information provided on NHC [23].

The 5-Star Nursing Homes Quality Rating System for Nursing Homes is based on data from the Minimum Data Set (MDS) and includes several quality measures (QMs) derived from it. Concerns have been raised about the effectiveness of the 5-Star Quality Rating System for nursing homes because of its reliance on the MDS. The MDS has several limitations, including inconsistent reporting due to ambiguous instructions and subjective items [24]. In addition, comparing across facilities proves difficult when the QMs emphasize rare events. Such events usually lead to large SEs and large CIs, making it challenging to assess true quality differences [24]. The QMs are assumed to be linear; however, surprisingly, they are sometimes nonlinear [24,25]. The MDS QMs are also presumed to use the complete spectrum of possible values even in situations that deviate from medical norms, such as pressure ulcer rates below 2% [24,26]. Furthermore, ascertainment bias, a type of detection bias caused by inadequate recording of relevant QMs, and substantial interrater variability often impact the reliability of MDS data [24].

Additionally, nursing home studies face other challenges beyond those associated with the sector’s reliance on the MDS. One problem is the absence of resident-level data, which impedes the establishment of causality. Moreover, endogeneity is inadequately controlled due to the nonindependence of dependent variables and error terms, which leads to biased results [27-29]. Additionally, the omission of risk adjustment for relevant confounders may make it impossible to capture any associations between a confounder and the other variables [27,30]. In this study, we used a panel dataset to evaluate the effectiveness of several nursing home QMs in explaining nursing home QoC in terms of COVID-19 health outcomes. We achieved this by regressing COVID-19 outcomes measured in the US nursing homes during the period May 2020 to June 2023 on nursing home QMs, classified using the Donabedian structure-process-outcome (SPO) framework, which are used in constructing the 5-Star Quality Rating System for nursing homes. Our research contributes to the literature in several ways. First, most scholars who used COVID-19 health outcomes have so far considered only a limited number of nursing home QMs. Moreover, they have relied on small sample sizes or used cross-sectional data over longitudinal data. We addressed these limitations by using a panel dataset of COVID-19 data for the nursing homes in the United States collected during the period May 2020 to July 2023. Second, to tackle the challenge of large SEs associated with rare events, we opted for a larger sample size, thus enhancing statistical power and refining our estimates. Third, to mitigate the concern of inadequate control of endogeneity, we used hierarchical generalized linear mixed effects modeling.

We hypothesized that nursing homes with better structural quality (eg, greater staff availability and better skill mix), better process quality (eg, lower restraint use and higher vaccination rates), and better outcome quality (eg, lower number of residents with pressure ulcers and lower number of resident falls) experienced better COVID-19 performance in terms of resident and staff infections and deaths. An empirical analysis of this issue offers fresh perspectives that were previously unavailable from existing research.

## Methods

### Data Sources

To test the study’s hypotheses, we used a panel dataset created using 3 major publicly available data sources: COVID-19 nursing home data, Payroll-Based Journal data, and nursing home QM snapshots.

COVID-19 health outcomes for the period May 2020 to June 2023 were obtained from COVID-19 nursing home data. The latter were obtained as a single dataset containing weekly summaries of resident and staff infections and deaths. Payroll-Based Journal data are collected quarterly and offer daily summaries of employee weekly hours for different staff, including nursing, non-nursing, employee, and contract staff. Nursing home QM snapshots include monthly summaries of QMs obtained from multiple data sources, such as the MDS, claims, penalties, provider information, survey summaries, and vaccination data.

The data were aggregated to create a unified dataset. Data aggregation was performed using 2 unique identifiers: one identified the facility (ie, the federal provider number), whereas the other identified the period (ie, week, month, or quarter). All statistical analyses were performed using R version 4.0.0 or higher (R Foundation for Statistical Computing) [31], and a 2-sided *P* less than .05 was considered statistically significant.

### Dependent Variables

The measures “residents weekly confirmed COVID-19,” “residents weekly COVID-19 deaths,” “staff weekly confirmed COVID-19,” and “staff weekly COVID-19 deaths” monitored the number of residents or staff who tested positive for COVID-19 and those who died due to COVID-19 in a particular week. These figures were reported by providers weekly from May 24, 2020, to June 6, 2023, with the data for the week ending May 25, 2020, potentially including reporting from January 1, 2020, to May 24, 2020.

In the analysis, we combined “residents weekly COVID-19 deaths” and “staff weekly COVID-19 deaths” into a single value representing “total (resident and staff) weekly COVID-19 deaths.” We adopted this approach because both variables were characterized by sparsity and a high number of zeros, and combining them in this manner increased statistical power and, subsequently, the variance for modeling of zero-inflated outcome variables [32].

### Independent Variables

The set of independent variables was categorized based on the Donabedian SPO framework. According to this framework, the quality of health care, including nursing home care, can be assessed at a facility based on 3 quality components: structure, process, and outcome. *Structure* refers to the settings where health care is provided (eg, buildings and technology infrastructure) and accessibility features. *Process* encompasses the actions taken in giving and receiving care, such as pain management, error prevention, and care follow-ups. *Outcome* indicates the consequences of the provided health care, including mortality rates, readmission rates, and functional status. The quality of health care is contingent upon the interplay of these categories, as Donabedian eloquently stated, “A good structure increases the likelihood of good process, and good process increases the likelihood of good outcomes” [27,33].

### Structural QMs

For our analysis, we used a comprehensive array of structural QMs encompassing various nursing home attributes (Textbox 1). To gauge the workforce dynamics, we factored in staffing hours, which were calculated separately for employees and contract staff and for nursing and non-nursing staff; we also examined turnover rates for nursing personnel, registered nurses, and non-nursing staff. Furthermore, we considered characteristics tied to the 5-Star Quality Rating System for nursing homes, including facility fines, penalties, and health and fire safety deficiencies, alongside organizational attributes, such as ownership type, provider category, special focus status, and the presence of resident and family councils, all of which contributed to our assessment of overall performance and stability.

Textbox 1. **Structural quality measures (count model predictors). “N” denotes a numeric variable, whereas “C” denotes a categorical variable. The number preceding “C” represents the number of levels in the categorical variable.**

Provider information model variables

- Percentage of occupied beds (N)
- Provider type (4C)
- Provider resides in hospital (2C)
- Days since approval to provide Medicare and Medicaid services (N)
- Continuing care retirement community (2C)
- Special focus status (2C)
- Abuse icon (2C)
- Most recent health inspection more than 2 years (2C)
- Provider changed owner in previous 12 months (2C)
- With a resident and family council (4C)

Penalties and staffing model variables

- Employee nursing total weekly hours (N)
- Employee non-nursing total weekly hours (N)
- Contract nursing total weekly hours (N)
- Contract non-nursing total weekly hours (N)

### Process QMs

An ample set of process QMs was considered (Textbox 2). Among these were factors pertinent to COVID-19, such as the number of residents hospitalized with confirmed cases and their vaccination status, along with weekly admissions of residents previously treated for the virus. The health care aspects included measures such as the percentage of long-stay residents appropriately receiving pneumococcal and influenza vaccines, the percentage of long-stay residents receiving antianxiety or hypnotic medications, those subjected to physical restraints, and those experiencing adverse effects of in-dwelling catheters. For short-stay residents, the metrics focused on appropriate pneumococcal and influenza vaccination rates and the percentage of residents receiving newly prescribed antipsychotic medications.

Textbox 2. **Process quality measures (count model predictors). “N” denotes a numeric variable.**

Process model variables

- Residents’ weekly COVID-19 admissions (N)
- Residents hospitalized with confirmed COVID-19 (N)
- Residents hospitalized with confirmed COVID-19 and up to date with vaccines (N)
- Percentage of current residents up to date with COVID-19 vaccines (N)
- Percentage of current health care personnel up to date with COVID-19 vaccines (N)
- Percentage of long-stay residents assessed and appropriately given the pneumococcal vaccine (N)
- Percentage of long-stay residents assessed and appropriately given the seasonal influenza vaccine (N)
- Percentage of long-stay residents who have received an antianxiety or hypnotic medication (N)
- Percentage of long-stay residents who have received an antipsychotic medication (N)
- Percentage of long-stay residents who are physically restrained (N)
- Percentage of long-stay residents with catheters inserted and left in their bladders (N)
- Percentage of short-stay residents assessed and appropriately given the pneumococcal vaccine (N)
- Percentage of short-stay residents who have received a newly prescribed antipsychotic medication (N)
- Percentage of short-stay residents assessed and appropriately given the seasonal influenza vaccine (N)

### Outcome QMs

The outcome QMs used for the analysis encompassed various measures of resident care and well-being (Textbox 3). Long-stay measures focusing on depressive symptoms, weight loss, deteriorating mobility, increased need for assistance with daily activities, urinary tract infections (UTIs), and loss of bowel or bladder control were included. Short-stay measures evaluated functional improvements in mobility, outpatient emergency department visits, and rehospitalizations within 30 days of admission.

Textbox 3. **Outcome quality measures (count model predictors). “N” denotes a numeric variable.**

Outcome model variables

- Number of residents with a new positive COVID-19 test result (N)
- Number of staff and personnel with a new positive COVID-19 test result (N)
- Percentage of SNF residents with new or worsened pressure ulcers (N)
- Percentage of high-risk long-stay residents with pressure ulcers (N)
- Percentage of long-stay residents who have experienced one or more falls with major injury (N)
- Percentage of long-stay residents who have depressive symptoms (N)
- Percentage of long-stay residents who have lost too much weight (N)
- Percentage of long-stay residents whose ability to move independently has worsened (N)
- Percentage of long-stay residents whose need for help with daily activities has increased (N)
- Percentage of long-stay residents with urinary tract infections (N)
- Percentage of low-risk long-stay residents who lose control of their bowels or bladders (N)
- Percentage of short-stay residents who have made improvements in function (N)
- Percentage of short-stay residents who have had an outpatient emergency department visit (N)
- Percentage of short-stay residents who have been rehospitalized after a nursing home admission (N)

Penalties and staffing model variables

- Total fines (N)
- Total amount (N)
- Total penalties (N)
- Total days (N)
- Number of facility-reported incidents (N)
- Total nursing staff turnover (N)
- Registered nurse turnover (N)
- Number of administrators who have left the nursing home (N)

Surveys model variables

- Total health deficiencies inspection cycle 1 (N)
- Total health deficiencies inspection cycle 2 (N)
- Total health deficiencies inspection cycle 3 (N)
- Total fire safety deficiencies inspection cycle 1 (N)
- Total fire safety deficiencies inspection cycle 2 (N)
- Total fire safety deficiencies inspection cycle 3 (N)
- Total weighted health survey score (N)

### Data Cleaning

**Figure 1.**
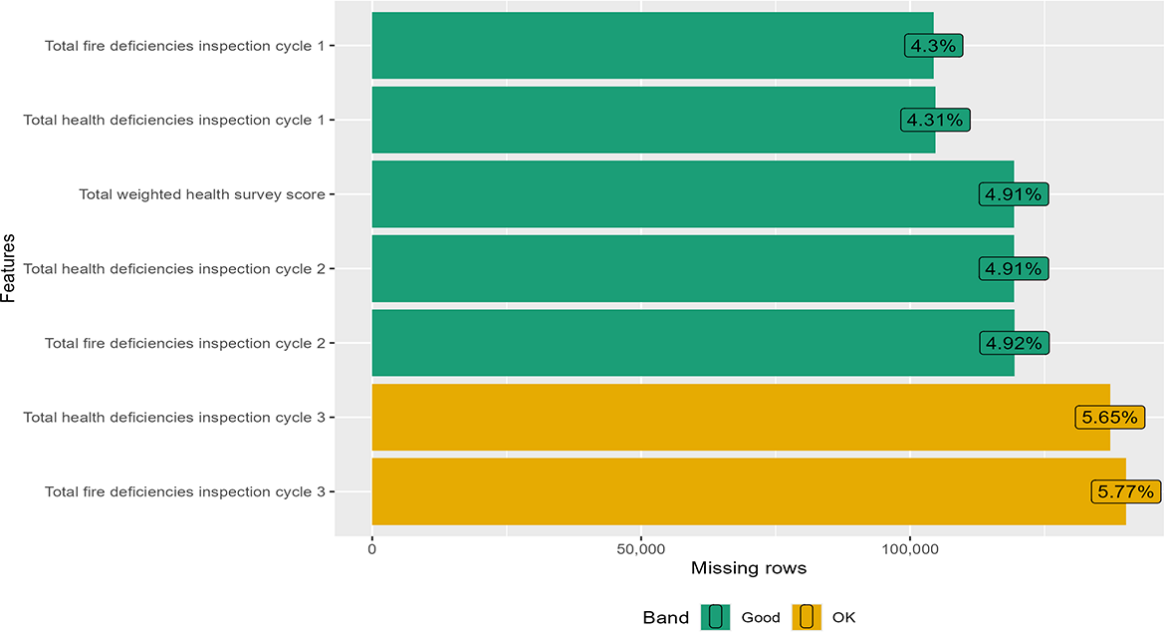
Missing value profile of the structural quality measures: surveys.

Prior to analysis, we removed all the observations in the dataset that contained a flag for either incorrect data or substandard data. This flagging was part of the Centers for Disease Control and Prevention’s quality assurance check, conducted by CMS on 8 data fields to ensure they did not contain implausible or erroneous values. Our decision to exclude flagged observations mirrors those made in prior research in the field [34]. Removing flagged observations eliminated 77,684 observations, resulting in a dataset containing 2,429,283 observations. The latter were used in the analysis after imputing with random forest. The missing value profiles of the variables are shown below (Figures1^5^).

**Figure 2.**
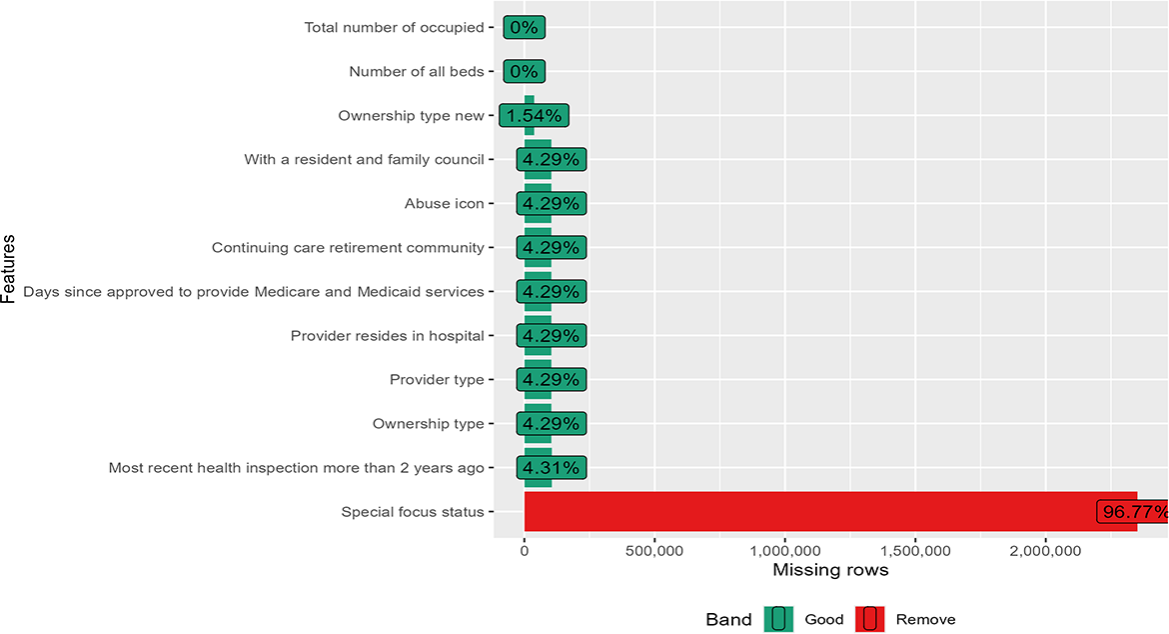
Missing value profile of the structural quality measures: provider information.

**Figure 3.**
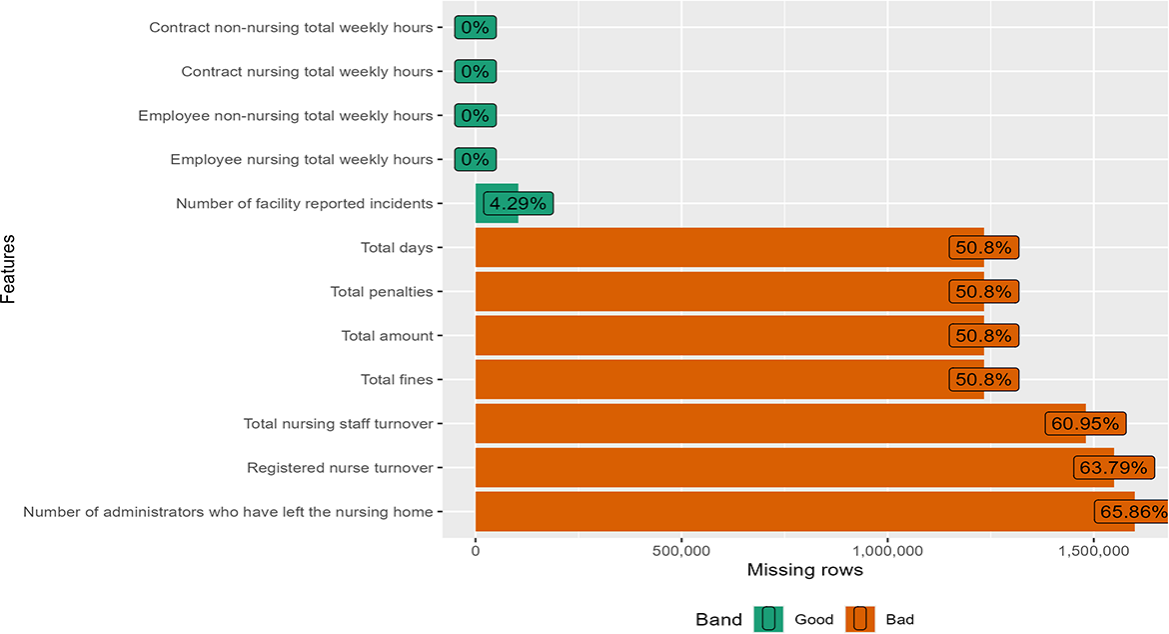
Missing value profile of the structural quality measures: penalties and staffing.

**Figure 4.**
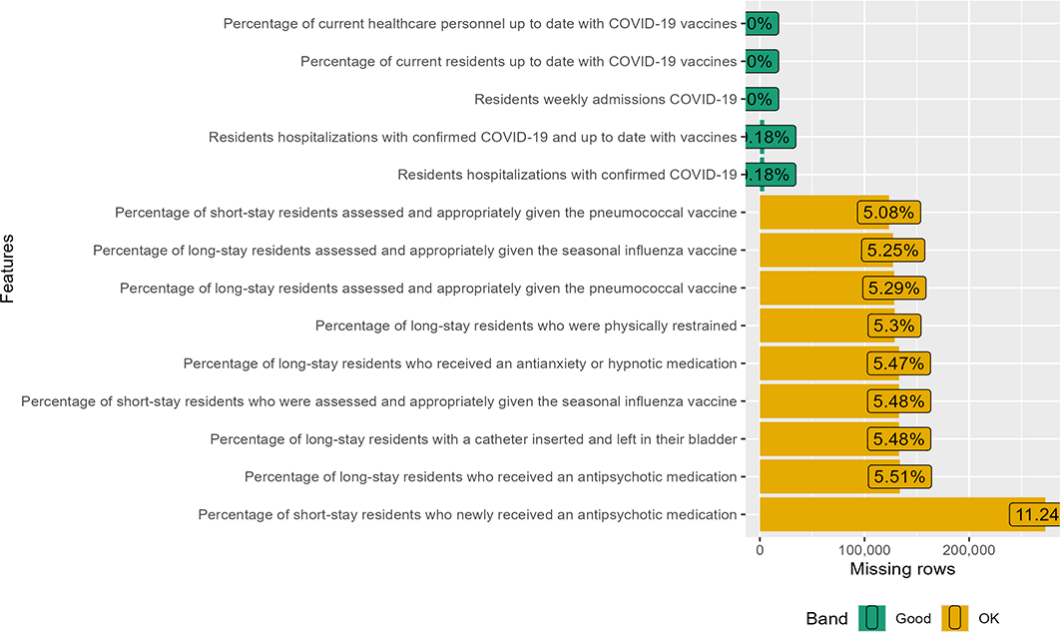
Missing value profile of the process quality measures.

**Figure 5.**
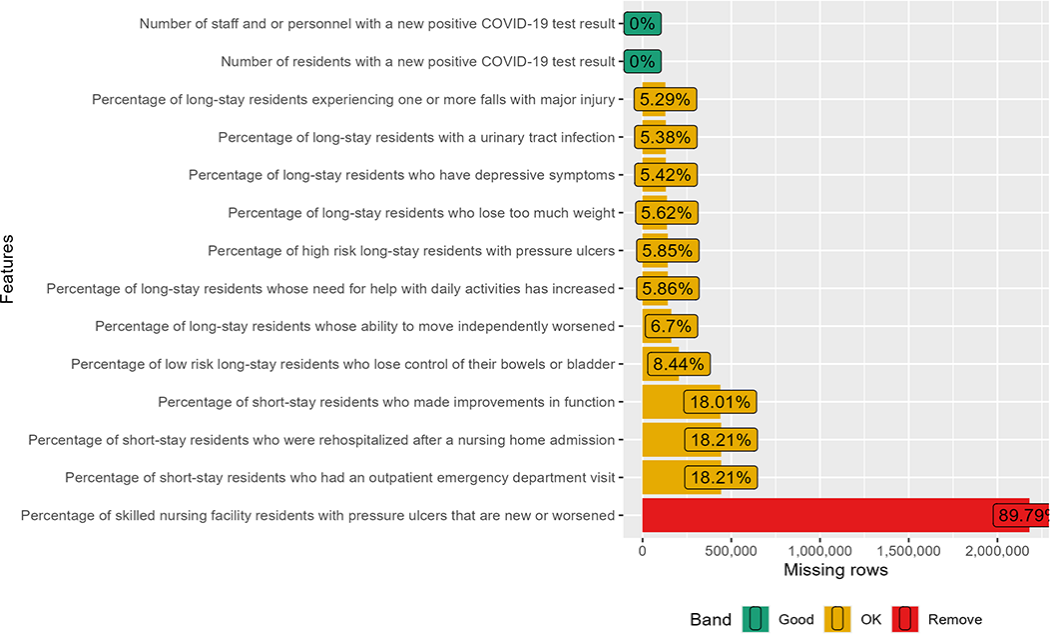
Missing value profile of the outcome quality measures.

### Missing Value Imputation

When addressing the presence of missing values in our dataset, we made the deliberate choice to impute rather than exclude them, which aligned with the research goals and the nature of the missing data. In our study, we assumed that the missing data were missing at random (MAR). The MAR mechanism assumes that the probability of missingness depends on the observed data but not on the missing data [35]. As the nursing home data are aggregated at the institution level and include all institutions nationwide, missingness is likely related to differences in administrative or operational factors, such as staffing levels, documentation practices, reporting systems, or governance policies at the state, county, or local levels. We opted for random forest imputation, as it handles various data types seamlessly; it also excels in managing complex interactions and multicollinearity while efficiently scaling to accommodate large datasets. The implementation of random forest in the R package *missranger* [36] resulted in a reduction of up to 50% of imputation errors, and the average proportional deviation in out-of-bag imputation errors remained within the 10% to 15% range. All the variables (except the outcome variables) were imputed using the random forest algorithm. Although the best method to address MAR missingness is to perform multiple imputation [37], we were unable to adopt this approach due to time and resource constraints. The specifications used for random forest imputation are presented in Table 1.

**Table 1.** Specifications used for random forest imputation.

Parameter	Value
Predictive mean matching	5
Maximum iterations	20
Number of trees	20
Maximum depth	8

### Data Modeling

Data Modeling

Using the imputed dataset, the variable called “percentage of occupied beds” was created using “total number of occupied beds” and “number of all beds.” A total of 10 observations from the calculation resulted in a not-a-number error and were excluded. All the numeric variables were standardized to have a mean of 0 and an SD of 1. All the unordered categorical variables or nominal variables were specified using sum contrasts. For ordered categorical variables or ordinal variables, we used orthogonal polynomial contrasts, and we standardized them onto a uniform scale. Consequently, each contrast column had a mean of 0 and an SD of 1. This approach allowed us to fit the regression models within a standardized framework and easily compare different model coefficients.

Furthermore, all unordered categorical variables containing more than 2 levels were binary encoded before being specified using sum contrasts. Binary encoding is a machine learning technique that combines hash encoding and one-hot encoding. First, all levels of an unordered categorical variable are expressed using a unique numeric value. Then, the numbers are transformed into a unique combination of zeros and ones, making it efficient to store data that exhibit high cardinality.

The outcome variables were characteristic of count data and exhibited properties such as nonnegativity, integer values, positive skewness, and a greater prevalence of lower count values. Equidispersion assessment revealed significant overdispersion, which rendered the use of a traditional Poisson regression model unsuitable. Therefore, we used a negative binomial regression model. Unlike the Poisson model, the negative binomial model accounts for overdispersion with quadratic parameterization. The presence of excess zeros led to the implementation of a zero-inflated negative binomial (ZINB) model, and the panel structure of the dataset resulted in the specification of a mixed effects model. However, specifying a ZINB model requires the presence of zero inflation at all levels of the grouping variables (ie, states, counties, and facilities). Some of the levels did not have zero inflation; they had zero deflation. On the basis of recommendations in the literature [38], the final model we specified was a truncated ZINB mixed effects model.

We estimated multiple truncated ZINB models, specifying different outcome variables. To model the outcomes “residents weekly confirmed COVID-19” and “staff weekly confirmed COVID-19,” the zero inflation (ZI) component of the model included factors influencing initial susceptibility to COVID-19 [39]. These included facility-level characteristics, such as bed size, as well as social determinants of health attributes, including urbanicity, political affiliation of the geographic location, median household income, and local COVID-19 vaccination coverage. To model the outcome “total weekly COVID-19 deaths,” the ZI component of the model included factors influencing the possibility of death after COVID-19 had been contracted, such as lagged weekly resident confirmed COVID-19 cases per 1000 residents and lagged county confirmed COVID-19 cases per 1000 people. We assumed that newly infected residents (ie, infected at 1 time point before) had a higher likelihood of death compared to those who were not newly infected (ie, infected more than one time point before). Our assumption was informed by prior research, indicating that individuals who test positive for COVID-19 typically do not begin to develop detectable antibodies until approximately 1 week after the onset of symptoms [40]. Both lagged variables were calculated for a lag of 1. An overview of the outcome variables and their associated ZI model predictors is presented in (Textbox 4).

Textbox 4. **Model outcomes and ZI model predictors. Letter “N” denotes a numeric variable, whereas “C” denotes a categorical variable. The number preceding “C” represents the number of levels in the categorical variable.**

Residents weekly confirmed COVID-19

- Bed size (3C)
- Urban binary (2C)
- Democrat or Republican (2C)
- Median household income dollars inflation adjusted to data file year ACS (American Community Survey) 2016-2020 (N)
- Partially or fully vaccinated percent (N)

Staff weekly confirmed COVID-19

- Bed size (3C)
- Urban binary (2C)
- Democrat or Republican (2C)
- Median household income dollars inflation adjusted to data file year ACS 2016-2020 (N)
- Partially or fully vaccinated percent (N)

Total weekly COVID-19 deaths

- Lagged weekly resident confirmed COVID-19 cases per 1000 residents (N)
- Lagged county confirmed cases USA Facts new (N)

The model included random slopes and random intercepts for 3 levels of nesting, that is, nursing home facilities nested within counties nested within states. An autoregressive process of order-1 covariance structure was selected to model the correlations among time points for the same individual. The truncated negative binomial distribution with quadratic parameterization implemented in the R package *glmmTMB* [41] under the family “truncated_nbinom2” was used.

Mathematically, the model can be expressed as follows:


$$\mu =E(\text{count}|u,NSZ)=\exp(\beta_{0}+\beta_{i}x_{i}+u)\;\forall \;\text{count}§gt;0$$

$$u\sim N(0,\sigma_{u}^{2})$$

$$\sigma^{2}=Var\left(count|u,\;NSZ\right)=\;\mu \left(1+\frac{\mu }{\theta }\right)\forall \;count>0$$

$$logit\left(p\right)=log\left(\frac{p}{1-p}\right)=\beta_{0}^{\left(zi\right)}+\beta_{i}^{\left(zi\right)}x_{i}$$

where subscript I denotes the time point, u denotes the random effects terms in the model, NSZ denotes the event “nonstructural zeros,” *P*=1−Pr(NSZ) is the ZI probability, and θ is the dispersion parameter specific to the family “nbinom2.”

**Figure 6.**
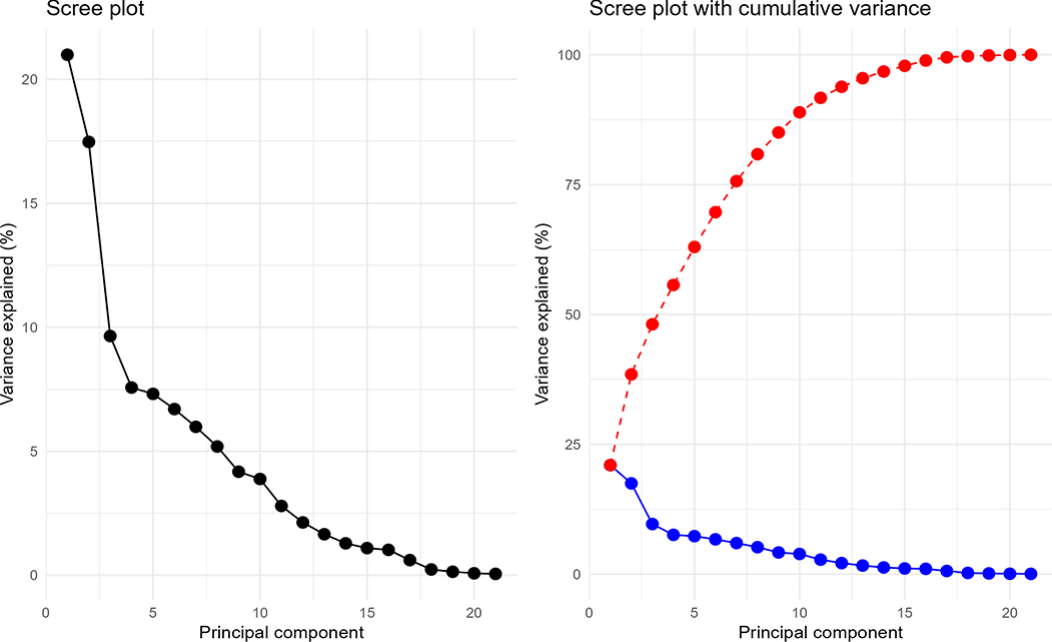
Variation of the principal components.

For each outcome variable, we created 5 base models. Each model contained a different subset of nursing home QMs, namely, outcome QMs, process QMs, and structural QMs (surveys, provider information, and penalties and staffing). Using a subset of significant predictors (30/58) from the 5 base models, we performed principal component analysis (PCA) for dimensionality reduction on the mean-aggregated dataset, where data were averaged across repeated measures for each entity. Using the loadings (eigenvectors) from this PCA, we calculated principal component scores for the complete dataset, including all the repeated measures. Using the PCA scores as predictors, we created 2 predictive models for the states and the components of the 5-Star Quality Rating System of nursing homes. The first 8 principal components explained 80.86% of the variation present in 30 significant predictors (Figure 6).

### Model Diagnostics

We performed model fit assessment through a combination of graphical diagnostics, goodness-of-fit statistics, and residual analysis. First, observed versus predicted count distributions were compared using histograms to visually assess the model’s ability to accurately predict both zero and nonzero values. Second, goodness-of-fit statistics were computed, including the log-likelihood, Akaike Information Criterion, Bayesian Information Criterion, and deviance to evaluate and compare across statistical models. We selected several goodness-of-fit measures to achieve a more robust evaluation of model performance. Third, the Vuong test was applied to compare the ZINB model against standard negative binomial and Poisson models to determine whether the inclusion of a ZI component was statistically justified. Finally, residual diagnostics were performed using the R package *DHARMa* [42], and goodness-of-fit was evaluated through nonparametric, simulation-driven methods. *DHARMa* provided an assessment of outliers, over- or underdispersion, ZI, and the independence of observations by simulating new response data, computing empirical cumulative density functions, and defining residuals based on observed versus simulated values, ensuring a robust model adequacy assessment.

### Ethical Considerations

This study is exempt from the institutional review board, as it involved only the use of publicly available data and did not involve human participants, in accordance with 45 CFR 46.102(e). No private, identifiable information was collected or analyzed.

## Results

For the models involving outcome QMs as predictors, the number of new COVID-19 cases among residents (β=0.46, SE 0.002; β=0.02, SE 0. 001; and β=0.02, SE 0.003) and staff (β=0.01, SE 0.001; β=0.51, SE 0.002; and β=0.05, SE 0.004) were highly significant, with all the outcomes at the significance levels of *P*<.001 and *P*<.001 respectively. The percentage of long-stay residents with UTIs was significant, with staff infections as the outcome (β=−0.004, SE 0.002) at the significance level of *P*<.10 (*P*=0.06), resident infections as the outcome (β=−0.01, SE 0.003) at the significance level of *P*<.001, and total deaths as the outcome (β=−0.03, SE 0.009) at the significance level of *P*<.01 (*P*=.001).

For the models involving process QMs as predictors, the indicator concerning residents’ weekly COVID-19 admissions (β=0.05, SE 0.003; β=0.04, SE 0.001; and β=0.048, SE 0.002) was significant, with all the outcomes at the significance level of *P*<.001.

Residents’ hospitalizations with confirmed COVID-19 (β=0.03, SE 0.002 and β=0.02, SE 0.001), the percentage of current residents up to date with COVID-19 vaccines (β=0.06, SE 0.007 and β=0.02, SE 0.005), and the percentage of long-stay residents assessed and appropriately given the seasonal influenza vaccine (β=0.02, SE 0.005 and β=0.04, SE 0.01) were significant, with at least two of the outcomes at the significance levels of *P*<.001, *P*<.001, and *P*<.001, respectively.

Residents hospitalized with confirmed COVID-19 and up to date with vaccines (β=0.03, SE 0.005 and β=0.05, SE 0.02) and the percentage of long-stay residents with catheters inserted and left in their bladders (β=−0.05, SE 0.005; β=−0.01, SE 0.003; and β=−0.02, SE 0.009) were significant, with at least two of the outcomes at the significance level of *P*<.001 and *P*<.05, respectively.

For the models involving structural QMs (surveys) as predictors, total health deficiencies inspection cycle 2 (β=0.05, SE 0.005 and β=0.01, SE 0.004) was significant, with at least two outcomes at the *P*<.001 significance level.

Total health deficiencies inspection cycle 1 (β=0.04, SE 0.01 and β=0.01, SE 0.004), total health deficiencies inspection cycle 3 (β=0.04, SE 0.01 and β=0.01, SE 0.004), and total fire safety deficiencies inspection cycle 3 (β=0.02, SE 0.01 and β=0.01, SE 0.004) were significant, with at least one of the outcomes at the significance level of *P*<.001, *P*<.05, and *P*<.05, respectively.

For the models involving structural QM (provider information) as predictors, percentage of occupied beds (β=0.04, SE 0.005; β=−0.06, SE 0.004; and β=−0.25, SE 0.01), provider resides in hospital (β=0.19, SE 0.02; β=0.07, SE 0.01; and β=0.13, SE 0.03), and days since approval to provide Medicare and Medicaid services (β=0.08, SE 0.005; β=0.02, SE 0.005; and β=0.04, SE 0.01) were significant, with all outcomes at the *P*<.001, *P*<.001, and *P*<.001 significance levels, respectively.

Continuing care retirement community (β=0.08, SE 0.009 and β=0.06, SE 0.02) and at least one of the 2 binary components of provider type (component 1: β=0.26, SE 0.03; β=0.08, SE 0.03; and β=0.25, SE 0.06), with a resident and family council (component 1: β=0.02, SE 0.005; β=0.05, SE 0.01 and component 2: β=0.08, SE 0.01; β=0.03, SE 0.01; and β=0.16, SE 0.03), and new ownership type (component 2: β=0.05, SE 0.01 and β=−0.07, SE 0.01) were significant, with at least two of the outcomes at the *P*<.001, *P*<.001, *P*<.001, *P*<.001, and *P*<.001 significance level.

For the models involving structural QMs (penalties and staffing) as predictors, all the staffing variables, namely employee nurse total weekly hours (β=0.12, SE 0.006; β=0.09, SE 0.005; and β=0.06, SE 0.01), employee nonnursing total weekly hours (β=−1.06, SE 0.006; β=−0.09, SE 0.005; and β=−0.05, SE 0.01), contract nurse total weekly hours (β=0.1, SE 0.004; β=0.08, SE 0.003; and β=0.10, SE 0.008), and contract nonnursing total weekly hours (β=−0.1, SE 0.01; β=−0.1, SE 0.004; and β=−0.04, SE 0.01) were significant, with all the outcomes at the *P*<.001 significance level, respectively.

Component 3 of provider state (component 3: β=−0.23, SE 0.05; β=−0.23, SE 0.05; and β=−0.20, SE 0.06) was significant at the *P*<.001 significance level. None of the components of the provider county achieved a high level of significance.

The linear (β=0.08, SE 0.008 and β=0.05, SE 0.006), quadratic (β=0.02, SE 0.005 and β=0.02, SE 0.004), and cubic (β=0.03, SE 0.005 and β=0.02, SE 0.003) components of the health inspection rating were significant, with resident infections and staff infections as outcomes at the *P*<.001, *P*<.001, and *P*<.001 significance levels, respectively. However, only the quartic (β=−0.0156, SE 0.0079) component of the health inspection rating was significant, with total deaths as the outcome at the *P*<.05 (*P*=.048) significance level.

The QM rating had significant linear (β=−0.02, SE 0.01) and cubic components (β=−0.01, SE 0.005) in the model, with resident infections as the outcome at the *P*<.05 (*P*=.002) and *P*<.05 (*P*=.045) significance levels, respectively. In addition to the linear (β=0.0164, SE 0.0055) and cubic (β=−0.009, SE 0.003) components, the quartic (β=−0.006, SE 0.003) component was significant at the *P*<.05 (*P*=.003), *P*<.05 (*P*=.01), and *P*<.05 (*P*=.04) significance levels, respectively, in the model with staff infections as the outcome. The quadratic (β=−0.02, SE 0.01) and cubic (β=0.02, SE 0.01) components were significant at the *P*<.05 (*P*=.04) and *P*<.05 (*P*=.045) significance levels, respectively, in the model with total deaths as the outcome.

Staffing rating had a significant linear (β=0.02, SE 0.005) component in the models with staff infections and total deaths as the outcomes at the *P*<.001 significance level. The model with resident infections as the outcome only showed a significant cubic (β=−0.008, SE 0.004) trend at the *P*<.1 (*P*=.06) significance level. In the model with total deaths as the outcome, both the linear (β=0.12, SE 0.01) and quadratic (β=−0.05, SE 0.01) components were significant at the *P*<.001 and *P*<.001 significance levels, respectively, and the quartic (β=−0.01, SE 0.007) component was significant at the *P*<.1 (*P*=.05) significance level.

The discussed results are summarized in Tables2  3. Multimedia Appendix 1, containing the complete model results in the original (log) scale, and Multimedia Appendix 2, containing the visualization of their 95% CIs in the response (exponent) scale, are provided for further reference.

**Table 2.** Model results for base models. The table displays variables that were significant when regressed on more than 1 outcome. All estimates are presented on the original (log) scale and are conditioned on the variables included in the conditional model and the model random effects. Complete results are provided in Multimedia Appendix 1 and displayed graphically in Multimedia Appendix 2.

Variable	Model 1: resident infections, estimate (SE)	*P* value	Model 2: staff infections, estimate (SE)	*P* value	Model 3: total deaths, estimate (SE)	*P* value
Intercept	4.60 (0.05)	<.001	3.52 (0.04)	<.001	3.89 (0.06)	<.001
Number of residents with a new positive COVID-19 test result	0.46 (0.002)	<.001	0.02 (0.001)	<.001	0.02 (0.003)	<.001
Number of staff and/or personnel with a new positive COVID-19 test result	0.01 (0.001)	<.001	0.51 (0.002)	<.001	0.05 (0.004)	<.001
Percentage of long-stay residents whose ability to move independently worsened	0.01 (0.003)	.02	0.01 (0.003)	.007	−0.02 (0.01)	.22
Percentage of long-stay residents with a urinary tract infection	−0.02 (0.003)	<.001	−0.004 (0.002)	.06	−0.03 (0.01)	.001
Intercept	4.28 (0.08)	<.001	3.31 (0.06)	<.001	3.37 (0.06)	<.001
Residents weekly admissions COVID-19	0.05 (0.003)	<.001	0.04 (0.001)	<.001	0.05 (0.002)	<.001
Residents hospitalizations with confirmed COVID-19	0.03 (0.002)	<.001	0.02 (0.001)	<.001	0.01 (0.004)	.26
Residents hospitalizations with confirmed COVID-19 and up to date with vaccines	0.0003 (0.002)	.92	0.03 (0.005)	<.001	0.05 (0.02)	.001
Percentage of current residents up to date with COVID-19 vaccines	−0.06 (0.01)	<.001	−0.02 (0.005)	<.001	−0.10 (0.03)	.002
Percentage of current health care personnel up to date with COVID-19 vaccines	0.03 (0.01)	<.001	0.01 (0.004)	.04	−0.001 (0.03)	.99
Percentage of long-stay residents assessed and appropriately given the seasonal influenza vaccine	0.02 (0.01)	<.001	0.0001 (0.004)	.97	0.04 (0.01)	<.001
Percentage of long-stay residents who received an antipsychotic medication	0.04 (0.01)	<.001	0.01 (0.005)	.13	0.2 (0.1)	.06
Percentage of long-stay residents who were physically restrained	−0.02 (0.01)	<.001	−0.002 (0.004)	.70	.02 (0.01)	.09
Percentage of long-stay residents with a catheter inserted and left in their bladder	−0.05 (0.005)	<.001	−0.01 (0.003)	.04	−0.02 (0.01)	.009
Percentage of short-stay residents who were assessed and appropriately given the seasonal influenza vaccine	−0.04 (0.01)	<.001	0.001 (0.01)	.85	−0.0004 (0.01)	.03
Intercept	4.70 (0.08)	<.001	3.53 (0.06)	<.001	3.86 (0.06)	<.001
Total health deficiencies inspection cycle	0.04 (0.01)	<.001	0.01 (0.005)	.009	0.02 (0.01)	.09
Total health deficiencies inspection cycle 2	0.05 (0.01)	<.001	0.01 (0.004)	<.001	0.01 (0.01)	.30
Total health deficiencies inspection cycle 3	0.04 (0.01)	<.001	0.01 (0.004)	.007	0.02 (0.01)	.10
Total fire deficiencies inspection cycle 3	0.02 (0.005)	<.001	0.01 (0.004)	.006	0.02 (0.01)	.06
Intercept	3.98 (0.08)	<.001	3.35 (0.06)	<.001	3.13 (0.09)	<.001
Percent of occupied beds	0.04 (0.01)	<.001	−0.06 (0.004)	<.001	−0.25 (0.01)	<.001
Provider type b1	0.26 (0.03)	<.001	0.08 (0.03)	.001	0.25 (0.06)	<.001
Provider type b2	−0.02 (0.02)	.07	−0.001 (0.02)	.01	−0.03 (0.05)	.09
Provider resides in hospital	0.18 (0.02)	<.001	0.07 (0.01)	<.001	0.13 (0.03)	<.001
Days since approved to provide Medicare and Medicaid services	0.08 (0.01)	<.001	0.02 (0.005)	<.001	0.04 (0.01)	<.001
Continuing care retirement community	0.08 (0.01)	<.001	−0.01 (0.01)	.43	0.06 (0.02)	<.001
Special focus status	−0.03 (0.004)	<.001	−0.003 (0.004)	.07	−0.01 (0.01)	.29
With a resident and family council b1	0.01 (0.01)	.11	0.02 (0.01)	<.001	0.05 (0.01)	<.001
With a resident and family council b2	0.08 (0.01)	<.001	0.03 (0.01)	.001	0.16 (0.03)	<.001
Ownership type new b1	0.01 (0.01)	<.001	0.02 (0.01)	.06	0.04 (0.01)	.002
Ownership type new b2	0.05 (0.01)	<.001	−0.07 (0.01)	<.001	−0.03 (0.02)	.14
Intercept	4.70 (0.08)	<.001	3.52 (0.06)	<.001	3.85 (0.06)	<.001
Total days	0.01 (0.01)	.08	0.01 (0.004)	.002	0.004 (0.01)	.72
Employee nursing total weekly hours	0.12 (0.01)	<.001	0.09 (0.01)	<.001	0.06 (0.01)	<.001
Employee nonnursing total weekly hours	−0.11 (0.01)	<.001	−0.09 (0.05)	<.001	−0.05 (0.01)	<.001
Contract nursing total weekly hours	0.10 (0.004)	<.001	0.08 (0.003)	<.001	0.10 (0.01)	<.001
Contract non-nursing total weekly hours	−0.10 (0.01)	<.001	−0.09 (0.004)	<.001	−0.04 (0.01)	<.001
Number of facility reported incidents	0.02 (0.01)	<.001	−0.001 (0.004)	.80	0.02 (0.01)	.06
Registered nurse turnover	0.01 (0.005)	.002	−0.01 (0.003)	.008	0.01 (0.01)	.09

**Table 3.** Model results for prediction models. The table displays variables that were significant when regressed on more than one outcome. All estimates are presented on the original (log) scale and are conditioned on the variables included in the conditional model and the model random effects. Complete results are provided in Multimedia Appendix 1 and displayed graphically in Multimedia Appendix 2.

Variable	Model 1: resident infections, estimate (SE)	*P* value	Model 2: staff infections, estimate (SE)	*P* value	Model 3: total deaths, estimate (SE)	*P* value
Intercept rating	4.36 (0.05)	<.001	2.97 (0.05)	<.001	3.45 (0.06)	<.001
PC^a^ rating	0.26 (0.01)	<.001	0.17 (0.004)	<.001	0.04 (0.01)	<.001
PC2 rating	0.59 (0.005)	<.001	0.53 (0.003)	<.001	0.02 (0.01)	<.001
PC3 rating	−0.06 (0.01)	<.001	−0.09 (0.004)	<.001	−0.01 (0.009)	.06
PC4 rating	0.12 (0.005)	<.001	0.16 (0.003)	<.001	−0.02 (0.01)	.003
PC5 rating	−0.005 (0.01)	.42	−0.06 (0.005)	<.001	0.03 (0.01)	.005
PC6 - rating	0.17 (0.01)	<.001	0.3 (0.04)	<.001	0.21 (0.01)	<.001
PC7 - rating	0.06 (0.01)	<.001	0.08 (0.004)	<.001	0.25 (0.01)	<.001
PC8 - rating	−0.09 (0.005)	<.001	−0.2 (0.004)	<.001	−0.08 (0.01)	<.001
Health inspection rating.l	0.08 (0.01)	<.001	0.05 (0.01)	<.001	0.02 (0.01)	.16
Health inspection rating.q	0.02 (0.01)	<.001	0.02 (0.004)	<.001	0.004 (0.01)	.67
Health inspection rating.c	0.03 (0.005)	<.001	0.02 (0.003)	<.001	0.01 (0.01)	.05
QM rating.l	−0.02 (0.01)	.002	0.02 (0.01)	.003	0.01 (0.01)	.54
QM rating.c	−0.01 (0.005)	.04	−0.01 (0.003)	.01	0.02 (0.01)	.05
Staffing rating.l	−0.01 (0.01)	.13	0.02 (0.005)	<.001	−0.12 (0.01)	<.001
Intercept - provider state	4.37 (0.06)	<.001	2.98 (0.06)	<.001	3.45 (0.07)	<.001
PC - provider state	−0.24 (0.004)	<.001	−0.15 (0.003)	<.001	−0.05 (0.01)	<.001
PC2 - provider state	0.59 (0.004)	<.001	0.53 (0.003)	<.001	0.03 (0.01)	<.001
PC3 - provider state	−0.08 (0.01)	<.001	−0.1 (0.004)	<.001	−0.01 (0.008)	.05
PC4 - provider state	−0.12 (0.005)	<.001	−0.16 (0.003)	<.001	0.02 (0.01)	<.001
PC5 - provider state	0.005 (0.01)	.74	−0.05 (0.005)	<.001	0.03 (0.01)	<.001
PC6 - provider state	0.16 (0.01)	<.001	0.29 (0.004)	<.001	0.19 (0.01)	<.001
PC7 - provider state	−0.08 (0.01)	<.001	−0.09 (0.004)	<.001	−0.26 (0.01)	<.001
PC8 - provider state	−0.09 (0.005)	<.001	−0.2 (0.004)	<.001	−0.07 (0.01)	<.001
Provider state b3	−0.23 (0.05)	<.001	−0.23 (0.05)	<.001	−0.2 (0.06)	<.001
Provider state b4	−0.01 (0.05)	.01	−0.03 (0.05)	.55	0.11 (0.06)	.07
Intercept - provider county	4.31 (0.07)	<.001	2.96 (0.07)	<.001	3.47 (0.08)	<.001
PC - provider county	−0.24 (0.004)	<.001	−0.15 (0.003)	<.001	−0.05 (0.01)	<.001
PC2 - provider county	0.59 (0.004)	<.001	0.53 (0.003)	<.001	0.03 (0.01)	<.001
PC3 - provider county	−0.08 (0.01)	<.001	−0.1 (0.004)	<.001	−0.01 (0.008)	.09
PC4 - provider county	−0.12 (0.005)	<.001	−0.16 (0.003)	<.001	0.02 (0.01)	<.001
PC5 - provider county	0.004 (0.01)	.52	−0.05 (0.005)	<.001	0.03 (0.01)	<.001
PC6 - provider county	0.16 (0.01)	<.001	0.29 (0.004)	<.001	0.19 (0.01)	<.001
PC7 - provider county	−0.08 (0.01)	<.001	−0.09 (0.004)	<.001	−0.26 (0.01)	<.001
PC8 - provider county	−0.09 (0.005)	<.001	−0.2 (0.004)	<.001	−0.07 (0.01)	<.001

a PC: principal component.

## Discussion

### Principal Findings

In this study, we used a panel dataset to evaluate the effectiveness of several nursing home QMs in explaining nursing home QoC in terms of COVID-19 outcomes. Staffing measures (*P*<.001 for all variables in all models), health deficiency scores (*P*<.001 for all variables in at least 1 model), COVID-19 hospitalizations (*P*<.001 for all variables in at least 2 models), and vaccinations (*P*<.001 for all variables in at least 2 models) exhibited meaningful relationships with the COVID-19 outcomes. The 5-Star Quality Rating System for nursing homes, Medicaid dependency, and ownership showed no clear relationships with the COVID-19 outcomes.

We found a significant association with the percentage of long-stay residents with UTIs. In a retrospective study, the authors evaluated UTI diagnoses and antibiotic prescriptions in 622 COVID-19 hospital ward patients, and they found that 61% of cases had probably been overdiagnosed [43]. Several researchers studying long-term care facilities have also found evidence of overdiagnosis in the form of inappropriate initiation of antimicrobial treatment in 37% to 61% of patients [43-49].

We also identified significant associations with the percentages of long-stay and short-stay residents assessed and appropriately given the seasonal influenza vaccine. A study compared COVID-19 and seasonal influenza patient groups and suggested that preexisting chronic respiratory conditions more strongly impacted the severity of seasonal influenza than that of COVID-19 [50]. It has also been reported that chronic lower respiratory tract diseases claim the third highest number of lives among adults aged >65 years [51-55]. Therefore, older individuals with comorbidities for both respiratory infections may exhibit stronger symptoms of seasonal influenza than COVID-19, demanding management through the administration of seasonal influenza vaccines.

A significant association was uncovered for the percentage of long-stay residents with catheters inserted and left in their bladders. In a recent systematic review of 67 studies, scholars found that catheter prevalence among nursing home residents varied between 2.2% and 36.4%, with a typical rate of 7.3% [56,57]. Catheter-associated UTIs comprise 32% of all health care–associated infections [57,58]. The fact that catheter use is frequently reported in long-term care residents and is a leading cause of UTI helps explain our results, which show significant associations with COVID-19 outcomes for both the percentage of long-stay residents with UTIs and the percentage of long-stay residents with catheters inserted and left in their bladders.

Our findings indicate a significant association between health deficiency variables and resident and staff COVID-19 cases. During the period 2013 to 2017, more than four-fifths of nursing homes in the United States engaged in deficient infection prevention and control practices, including those that substantially reduced the spread of COVID-19, such as proper hand washing and isolation procedures [21,59]. It is possible that these poor practices continued during the pandemic, making nursing home residents and staff more susceptible to COVID-19 infections.

Several scholars have reported that lower resident density, measured by the proxy variable of the percentage of occupied beds, is associated with a lower prevalence of COVID-19 [59-65]. Our study showed a similar association with resident infections but an inverse association with staff infections and total deaths. We found that hospital-based nursing homes reported fewer COVID-19 cases and deaths than nonhospital-based nursing homes, which is consistent with the results of a study by Tarteret et al [66]. Gorges and Konetzka [67] demonstrated facility-level differences in QoC based on the proportion of residents with Medicaid coverage at the facility. Compared to nursing homes accepting Medicaid only, nursing homes accepting Medicare showed clearly better COVID-19 outcomes in only 2 of 3 models. In 1 model, the difference was less clear due to overlapping 95% CIs.

Evidence from various studies suggests that for-profit nursing homes are more prone to COVID-19 outbreaks compared to nonprofit and government nursing homes [59,61,65,68]. However, some scholars have found no statistically significant link in this regard [34,60,66]. We did not notice a clear difference between for-profit, nonprofit, and government nursing homes. Furthermore, no clear difference in outcomes was observed between nursing homes with or without continuing care retirement communities, resident councils, family councils, or resident and family councils. While not a key characteristic, nursing homes newly approved to provide Medicare and Medicaid services had better COVID-19 outcomes compared to long-standing nursing homes. This could be because newer nursing homes underwent inspections more recently than previously approved homes as part of their approval process.

Inadequate nursing staff have been linked to a higher likelihood of COVID-19 outbreaks in some studies [59,62,64,65,68-70], but others indicate no significant connection [61,65,69,71]. Our findings show that increased staffing hours for nursing staff resulted in worse COVID-19 outcomes, whereas increased staffing hours for non-nursing staff resulted in better COVID-19 outcomes. We did not explore these associations further based on the types of nursing and nonnursing staff.

Most researchers have found no meaningful connection between nursing home overall quality rating and COVID-19 outcomes [34,60-62,69,72-79]. Similarly, most scholars have found no clear relationships between COVID-19 outcomes and 2 components of the 5-Star Quality Rating System for Nursing Homes, namely staffing rating and health inspection rating [61,64,70,73,78]. Our analysis did not reveal a clear relationship between the components of the 5-Star Quality Rating System for nursing homes and COVID-19 outcomes.

### Limitations

This study has some limitations. It only considered temporal autocorrelation, without addressing spatial autocorrelation, thus ignoring potential interdependencies across spatial units. Temporal autocorrelation assumes that changes over time are isolated within units, which is rarely the case in interconnected systems, such as health care networks. We also did not perform multiple dataset imputations, which could have resulted in less robust statistical analyses, as a single imputation set may not adequately reflect the variability inherent in missing data. By generating a range of plausible datasets, MIs allow for more accurate and reliable parameter estimates by accounting for the uncertainty around the imputed values. Future scholars should consider modeling both spatial and temporal autocorrelations, as well as performing MIs, to increase the validity and reliability of their findings.

### Conclusions

The evolution of quality in the nursing home industry is truly remarkable. However, sustained progress is essential to meet the needs of the growing older adult population. Although widely used in the nursing home industry, the 5-Star Quality Rating System for nursing homes is an unreliable performance measure. As demonstrated through the results of the study, it is hard to delineate nursing homes’ performance based on the star ratings, after accounting for the underlying QMs. Concerted efforts from lawmakers, policy makers, and lobbyists will be required to refine and enhance the measure, thereby ensuring its reliability and effectiveness.

## Supplementary material

10.2196/72770 Multimedia Appendix 1 Complete set of results tables for all models estimated in the study. Tables are organized by outcome variable and by model specification, distinguishing between base models using the original variables and final models incorporating principal component–derived variables, respectively.

10.2196/72770 Multimedia Appendix 2 Complete set of results displayed graphically. Bars denote the point estimates, while whiskers indicate the corresponding 95% CIs. Each figure is divided into 2 panels, representing the conditional model and the zero-inflation model, respectively.

## Abbreviations

CMS: Centers for Medicare and Medicaid Services
MAR: missing at random
MDS: Minimum Data Set
NHC: nursing home care
PCA: principal component analysis
QM: quality measure
QoC: quality of care
SPO: structure, process, or outcome
UTI: urinary tract infection
ZI: zero inflation
ZINB: zero-inflated negative binomial
