# Supplementary material for: Demystifying Quality Metrics and Unveiling the True Measure of Quality of Care in Nursing Homes: Mixed Effects Analysis
Source: JMIR Hum Factors. 2026 Jan 29;13:e72770. doi: 10.2196/72770 (PMC12854662; doi:10.2196/72770)
Supplement: Multimedia Appendix 1 [file humanfactors-v13-e72770-s001.docx]

**Table 1**

Resident infections (models 1 - 5 results in the original (log) scale)

|  | | | | | |  |
| --- | --- | --- | --- | --- | --- | --- |
|  | *Dependent variable:* | | | | |  |
|  |  | | | | |  |
|  | model1: outcome | model2: process | model3: structure (surveys) | model4: structure (provider info) | model5: structure (penalties, staffing) |  |
|  | (1) | (2) | (3) | (4) | (5) |  |
|  | | | | | |  |
| ***conditional model*** |  |  |  |  |  |  |
| intercept | 4.6017^***^ | 4.2836^***^ | 4.7004^***^ | 3.979^***^ | 4.7009^***^ |  |
|  | (0.0482) | (0.077) | (0.0757) | (0.0824) | (0.0754) |  |
| number_of_residents_with_a_new_positive_covid_19_test_result_out | 0.4609^***^ |  |  |  |  |  |
|  | (0.0015) |  |  |  |  |  |
| number_of_staff_and_or_personnel_with_a_new_positive_covid_19_test_result_out | 0.0148^***^ |  |  |  |  |  |
|  | (0.0012) |  |  |  |  |  |
| percentage_of_snf_residents_with_pressure_ulcers_that_are_new_or_worsened_out | -0.0036 |  |  |  |  |  |
|  | (0.0023) |  |  |  |  |  |
| percentage_of_high_risk_long_stay_residents_with_pressure_ulcers_out | -0.0026 |  |  |  |  |  |
|  | (0.0026) |  |  |  |  |  |
| percentage_of_long_stay_residents_experiencing_one_or_more_falls_with_major_injury_out | -0.0039 |  |  |  |  |  |
|  | (0.0026) |  |  |  |  |  |
| percentage_of_long_stay_residents_who_have_depressive_symptoms_out | 0.0083^**^ |  |  |  |  |  |
|  | (0.0028) |  |  |  |  |  |
| percentage_of_long_stay_residents_who_lose_too_much_weight_out | 0.0036 |  |  |  |  |  |
|  | (0.0025) |  |  |  |  |  |
| percentage_of_long_stay_residents_whose_ability_to_move_independently_worsened_out | 0.0079^*^ |  |  |  |  |  |
|  | (0.0033) |  |  |  |  |  |
| percentage_of_long_stay_residents_whose_need_for_help_with_daily_activities_has_increased_out | 0.0042 |  |  |  |  |  |
|  | (0.0031) |  |  |  |  |  |
| percentage_of_long_stay_residents_with_a_urinary_tract_infection_out | -0.0159^***^ |  |  |  |  |  |
|  | (0.0026) |  |  |  |  |  |
| percentage_of_low_risk_long_stay_residents_who_lose_control_of_their_bowels_or_bladder_out | -0.0055^*^ |  |  |  |  |  |
|  | (0.0027) |  |  |  |  |  |
| percentage_of_short_stay_residents_who_made_improvements_in_function_out | 0.0026 |  |  |  |  |  |
|  | (0.0025) |  |  |  |  |  |
| percentage_of_short_stay_residents_who_had_an_outpatient_emergency_department_visit_out | 6e-04 |  |  |  |  |  |
|  | (0.0025) |  |  |  |  |  |
| percentage_of_short_stay_residents_who_were_rehospitalized_after_a_nursing_home_admission_out | -0.0011 |  |  |  |  |  |
|  | (0.0024) |  |  |  |  |  |
| residents_weekly_admissions_covid_19_pro |  | 0.0521^***^ |  |  |  |  |
|  |  | (0.0027) |  |  |  |  |
| residents_hospitalizations_with_confirmed_covid_19_pro |  | 0.0275^***^ |  |  |  |  |
|  |  | (0.0017) |  |  |  |  |
| residents_hospitalizations_with_confirmed_covid_19_and_up_to_date_with_vaccines_pro |  | 3e-04 |  |  |  |  |
|  |  | (0.0024) |  |  |  |  |
| percentage_of_current_residents_up_to_date_with_covid_19_vaccines_pro |  | -0.0574^***^ |  |  |  |  |
|  |  | (0.0066) |  |  |  |  |
| percentage_of_current_healthcare_personnel_up_to_date_with_covid_19_vaccines_pro |  | 0.0294^***^ |  |  |  |  |
|  |  | (0.0056) |  |  |  |  |
| percentage_of_long_stay_residents_assessed_and_appropriately_given_the_pneumococcal_vaccine_pro |  | -0.001 |  |  |  |  |
|  |  | (0.0066) |  |  |  |  |
| percentage_of_long_stay_residents_assessed_and_appropriately_given_the_seasonal_influenza_vaccine_pro |  | 0.0222^***^ |  |  |  |  |
|  |  | (0.005) |  |  |  |  |
| percentage_of_long_stay_residents_who_received_an_antianxiety_or_hypnotic_medication_pro |  | 0.0236^***^ |  |  |  |  |
|  |  | (0.0058) |  |  |  |  |
| percentage_of_long_stay_residents_who_received_an_antipsychotic_medication_pro |  | 0.0441^***^ |  |  |  |  |
|  |  | (0.0054) |  |  |  |  |
| percentage_of_long_stay_residents_who_were_physically_restrained_pro |  | -0.0184^***^ |  |  |  |  |
|  |  | (0.0054) |  |  |  |  |
| percentage_of_long_stay_residents_with_a_catheter_inserted_and_left_in_their_bladder_pro |  | -0.049^***^ |  |  |  |  |
|  |  | (0.0048) |  |  |  |  |
| percentage_of_short_stay_residents_assessed_and_appropriately_given_the_pneumococcal_vaccine_pro |  | -0.0235^**^ |  |  |  |  |
|  |  | (0.0083) |  |  |  |  |
| percentage_of_short_stay_residents_who_newly_received_an_antipsychotic_medication_pro |  | 0.0184^***^ |  |  |  |  |
|  |  | (0.0044) |  |  |  |  |
| percentage_of_short_stay_residents_who_were_assessed_and_appropriately_given_the_seasonal_influenza_vaccine_pro |  | -0.0401^***^ |  |  |  |  |
|  |  | (0.0072) |  |  |  |  |
| total_health_deficiencies_inspection_cycle_1_str |  |  | 0.0414^***^ |  |  |  |
|  |  |  | (0.0059) |  |  |  |
| total_health_deficiencies_inspection_cycle_2_str |  |  | 0.045^***^ |  |  |  |
|  |  |  | (0.0054) |  |  |  |
| total_health_deficiencies_inspection_cycle_3_str |  |  | 0.0363^***^ |  |  |  |
|  |  |  | (0.0051) |  |  |  |
| total_fire_deficiencies_inspection_cycle_1_str |  |  | 0.0208^***^ |  |  |  |
|  |  |  | (0.005) |  |  |  |
| total_fire_deficiencies_inspection_cycle_2_str |  |  | 0.0067 |  |  |  |
|  |  |  | (0.005) |  |  |  |
| total_fire_deficiencies_inspection_cycle_3_str |  |  | 0.0214^***^ |  |  |  |
|  |  |  | (0.0049) |  |  |  |
| total_weighted_health_survey_score_str |  |  | 4e-04 |  |  |  |
|  |  |  | (0.0063) |  |  |  |
| percent_of_occupied_beds |  |  |  | 0.0396^***^ |  |  |
|  |  |  |  | (0.0051) |  |  |
| provider_type_str_b11 |  |  |  | 0.2561^***^ |  |  |
|  |  |  |  | (0.0265) |  |  |
| provider_type_str_b21 |  |  |  | -0.0404^+^ |  |  |
|  |  |  |  | (0.0225) |  |  |
| provider_resides_in_hospital_str1 |  |  |  | 0.1788^***^ |  |  |
|  |  |  |  | (0.015) |  |  |
| days_since_approved_to_provide_medicare_and_medicaid_services_str |  |  |  | 0.076^***^ |  |  |
|  |  |  |  | (0.0052) |  |  |
| continuing_care_retirement_community_str1 |  |  |  | 0.0848^***^ |  |  |
|  |  |  |  | (0.0085) |  |  |
| special_focus_status_str1 |  |  |  | -0.0342^***^ |  |  |
|  |  |  |  | (0.0044) |  |  |
| abuse_icon_str1 |  |  |  | -0.026^**^ |  |  |
|  |  |  |  | (0.0085) |  |  |
| most_recent_health_inspection_more_than_2_years_ago_str1 |  |  |  | 0.0063 |  |  |
|  |  |  |  | (0.0058) |  |  |
| provider_changed_ownership_in_last_12_months_str1 |  |  |  | -0.0334^*^ |  |  |
|  |  |  |  | (0.013) |  |  |
| with_a_resident_and_family_council_str_b11 |  |  |  | 0.0097 |  |  |
|  |  |  |  | (0.006) |  |  |
| with_a_resident_and_family_council_str_b21 |  |  |  | 0.0819^***^ |  |  |
|  |  |  |  | (0.0117) |  |  |
| ownership_type_new_str_b11 |  |  |  | 0.1043^***^ |  |  |
|  |  |  |  | (0.0066) |  |  |
| ownership_type_new_str_b21 |  |  |  | 0.0464^***^ |  |  |
|  |  |  |  | (0.0111) |  |  |
| total_fines_str |  |  |  |  | 0.0047 |  |
|  |  |  |  |  | (0.0046) |  |
| total_amount_str |  |  |  |  | 0.0097^*^ |  |
|  |  |  |  |  | (0.0044) |  |
| total_penalties_str |  |  |  |  | -0.0077 |  |
|  |  |  |  |  | (0.0054) |  |
| total_days_str |  |  |  |  | 0.0093^+^ |  |
|  |  |  |  |  | (0.0053) |  |
| emp_nurse_total_str |  |  |  |  | 0.1169^***^ |  |
|  |  |  |  |  | (0.0064) |  |
| emp_non_nurse_total_str |  |  |  |  | -0.1061^***^ |  |
|  |  |  |  |  | (0.0059) |  |
| ctr_nurse_total_str |  |  |  |  | 0.0963^***^ |  |
|  |  |  |  |  | (0.0039) |  |
| ctr_non_nurse_total_str |  |  |  |  | -0.0996^***^ |  |
|  |  |  |  |  | (0.005) |  |
| number_of_facility_reported_incidents_str |  |  |  |  | 0.0221^***^ |  |
|  |  |  |  |  | (0.005) |  |
| total_nursing_staff_turnover_str |  |  |  |  | 0.0025 |  |
|  |  |  |  |  | (0.0049) |  |
| registered_nurse_turnover_str |  |  |  |  | 0.0145^**^ |  |
|  |  |  |  |  | (0.0047) |  |
| number_of_administrators_who_have_left_the_nursing_home_str |  |  |  |  | 0.0109^**^ |  |
|  |  |  |  |  | (0.0034) |  |
|  |  |  |  |  |  |  |
|  |  |  |  |  |  |  |
|  |  |  |  |  |  |  |
|  |  |  |  |  |  |  |
|  |  |  |  |  |  |  |
|  |  |  |  |  |  |  |
|  |  |  |  |  |  |  |
|  |  |  |  |  |  |  |
|  |  |  |  |  |  |  |
|  |  |  |  |  |  |  |
|  |  |  |  |  |  |  |
|  |  |  |  |  |  |  |
|  |  |  |  |  |  |  |
|  |  |  |  |  |  |  |
|  |  |  |  |  |  |  |
|  |  |  |  |  |  |  |
|  |  |  |  |  |  |  |
|  |  |  |  |  |  |  |
|  |  |  |  |  |  |  |
|  |  |  |  |  |  |  |
|  |  |  |  |  |  |  |
|  |  |  |  |  |  |  |
|  |  |  |  |  |  |  |
|  |  |  |  |  |  |  |
|  |  |  |  |  |  |  |
|  |  |  |  |  |  |  |
|  |  |  |  |  |  |  |
|  |  |  |  |  |  |  |
|  |  |  |  |  |  |  |
|  |  |  |  |  |  |  |
|  |  |  |  |  |  |  |
|  |  |  |  |  |  |  |
|  |  |  |  |  |  |  |
|  |  |  |  |  |  |  |
|  |  |  |  |  |  |  |
|  |  |  |  |  |  |  |
|  |  |  |  |  |  |  |
|  | | | | | |  |
| ***zero-inflation model*** |  |  |  |  |  |  |
| intercept | 2.034^***^ | 2.034^***^ | 2.034^***^ | 2.034^***^ | 2.034^***^ |  |
|  | (0.0027) | (0.0027) | (0.0027) | (0.0027) | (0.0027) |  |
| urban_binary1 | 0.0971^***^ | 0.0971^***^ | 0.0971^***^ | 0.0971^***^ | 0.0971^***^ |  |
|  | (0.0024) | (0.0024) | (0.0024) | (0.0024) | (0.0024) |  |
| bed_size.L | 0.3455^***^ | 0.3455^***^ | 0.3455^***^ | 0.3455^***^ | 0.3455^***^ |  |
|  | (0.0026) | (0.0026) | (0.0026) | (0.0026) | (0.0026) |  |
| partially_of_fully_vaccinated_percent | -0.1515^***^ | -0.1515^***^ | -0.1515^***^ | -0.1515^***^ | -0.1515^***^ |  |
|  | (0.0019) | (0.0019) | (0.0019) | (0.0019) | (0.0019) |  |
| dem_gop1 | -0.043^***^ | -0.043^***^ | -0.043^***^ | -0.043^***^ | -0.043^***^ |  |
|  | (0.0023) | (0.0023) | (0.0023) | (0.0023) | (0.0023) |  |
| median_household_income_dollars_inflation_adjusted_to_data_file_year_acs_2016_2020 | -0.0025 | -0.0025 | -0.0025 | -0.0025 | -0.0025 |  |
|  | (0.002) | (0.002) | (0.002) | (0.002) | (0.002) |  |
|  | | | | | |  |
| Nobs | 2429283 | 2429283 | 2429283 | 2429273 | 2429283 |  |
| AIC | 3347408 | 3562647 | 3563609 | 3561991 | 3562915 |  |
| BIC | 3347764 | 3563003 | 3563876 | 3562346 | 3563245 |  |
| LogLik | -1673676 | -1781296 | -1781784 | -1780967 | -1781431 |  |
| Deviance | 3347352 | 3562591 | 3563567 | 3561935 | 3562863 |  |
|  | | | | | |  |
| *Note:* | ^+^p<0.1; ^*^p<0.05; ^**^p<0.01; ^***^p<0.001 | | | | | |

**Table 2**

**Resident infections (models 6 - 8 results in the original (log) scale)**

|  | | | |  |
| --- | --- | --- | --- | --- |
|  | *Dependent variable:* | | |  |
|  |  | | |  |
|  | Model6: five-star system estimates | Model7: state estimates | Model8: county estimates |  |
|  | (1) | (2) | (3) |  |
|  | | | |  |
| ***conditional model*** |  |  |  |  |
| intercept | 4.36^***^ | 4.3681^***^ | 4.3051^***^ |  |
|  | (0.0527) | (0.0621) | (0.0723) |  |
| health_inspection_rating.L | 0.0805^***^ |  |  |  |
|  | (0.0077) |  |  |  |
| health_inspection_rating.Q | 0.0174^***^ |  |  |  |
|  | (0.0051) |  |  |  |
| health_inspection_rating.C | 0.0308^***^ |  |  |  |
|  | (0.0045) |  |  |  |
| health_inspection_rating^4 | 9e-04 |  |  |  |
|  | (0.0039) |  |  |  |
| qm_rating.L | -0.0235^**^ |  |  |  |
|  | (0.0075) |  |  |  |
| qm_rating.Q | -0.0017 |  |  |  |
|  | (0.0057) |  |  |  |
| qm_rating.C | -0.0092^*^ |  |  |  |
|  | (0.0046) |  |  |  |
| qm_rating^4 | -0.0033 |  |  |  |
|  | (0.0039) |  |  |  |
| staffing_rating.L | -0.0102 |  |  |  |
|  | (0.0068) |  |  |  |
| staffing_rating.Q | -0.0012 |  |  |  |
|  | (0.0051) |  |  |  |
| staffing_rating.C | -0.0075^+^ |  |  |  |
|  | (0.0041) |  |  |  |
| staffing_rating^4 | -0.0023 |  |  |  |
|  | (0.0034) |  |  |  |
| PC1 | 0.2628^***^ | -0.2419^***^ | -0.2421^***^ |  |
|  | (0.0051) | (0.0042) | (0.0042) |  |
| PC2 | 0.5879^***^ | 0.5898^***^ | 0.5891^***^ |  |
|  | (0.0045) | (0.0044) | (0.0044) |  |
| PC3 | -0.0629^***^ | -0.0785^***^ | -0.0785^***^ |  |
|  | (0.0057) | (0.0054) | (0.0054) |  |
| PC4 | 0.1158^***^ | -0.1187^***^ | -0.1187^***^ |  |
|  | (0.0046) | (0.0046) | (0.0046) |  |
| PC5 | -0.0049 | 0.0049 | 0.0039 |  |
|  | (0.0061) | (0.0061) | (0.0061) |  |
| PC6 | 0.1704^***^ | 0.1637^***^ | 0.1635^***^ |  |
|  | (0.0052) | (0.0051) | (0.0051) |  |
| PC7 | 0.0631^***^ | -0.0759^***^ | -0.0762^***^ |  |
|  | (0.0053) | (0.0053) | (0.0053) |  |
| PC8 | -0.0944^***^ | -0.0876^***^ | -0.0876^***^ |  |
|  | (0.0047) | (0.0047) | (0.0047) |  |
| provider_state_b11 |  | -0.085 |  |  |
|  |  | (0.0532) |  |  |
| provider_state_b21 |  | -0.204^***^ |  |  |
|  |  | (0.0522) |  |  |
| provider_state_b31 |  | -0.2306^***^ |  |  |
|  |  | (0.0536) |  |  |
| provider_state_b41 |  | -0.1369^*^ |  |  |
|  |  | (0.0534) |  |  |
| provider_state_b51 |  | -0.1049^+^ |  |  |
|  |  | (0.0582) |  |  |
| provider_state_b61 |  | 0.0927 |  |  |
|  |  | (0.061) |  |  |
| provider_state_county_b11 |  |  | 0.016 |  |
|  |  |  | (0.0102) |  |
| provider_state_county_b21 |  |  | -0.0118 |  |
|  |  |  | (0.0102) |  |
| provider_state_county_b31 |  |  | 0.0049 |  |
|  |  |  | (0.0103) |  |
| provider_state_county_b41 |  |  | 0.0119 |  |
|  |  |  | (0.0103) |  |
| provider_state_county_b51 |  |  | -8e-04 |  |
|  |  |  | (0.0104) |  |
| provider_state_county_b61 |  |  | 0.006 |  |
|  |  |  | (0.0109) |  |
| provider_state_county_b71 |  |  | 0.0176 |  |
|  |  |  | (0.0122) |  |
| provider_state_county_b81 |  |  | 0.0187 |  |
|  |  |  | (0.0163) |  |
| provider_state_county_b91 |  |  | -0.008 |  |
|  |  |  | (0.0249) |  |
| provider_state_county_b101 |  |  | -0.0129 |  |
|  |  |  | (0.0363) |  |
| provider_state_county_b111 |  |  | -0.0069 |  |
|  |  |  | (0.0515) |  |
| provider_state_county_b121 |  |  | 0.0697 |  |
|  |  |  | (0.0681) |  |
|  |  |  |  |  |
|  | | | |  |
| ***zero-inflation model*** |  |  |  |  |
| intercept | 2.034^***^ | 2.034^***^ | 2.034^***^ |  |
|  | (0.0027) | (0.0027) | (0.0027) |  |
| urban_binary1 | 0.0971^***^ | 0.0971^***^ | 0.0971^***^ |  |
|  | (0.0024) | (0.0024) | (0.0024) |  |
| bed_size.L | 0.3455^***^ | 0.3455^***^ | 0.3455^***^ |  |
|  | (0.0026) | (0.0026) | (0.0026) |  |
| partially_of_fully_vaccinated_percent | -0.1515^***^ | -0.1515^***^ | -0.1515^***^ |  |
|  | (0.0019) | (0.0019) | (0.0019) |  |
| dem_gop1 | -0.0431^***^ | -0.0431^***^ | -0.0431^***^ |  |
|  | (0.0023) | (0.0023) | (0.0023) |  |
| median_household_income_dollars_inflation_adjusted_to_data_file_year_acs_2016_2020 | -0.0025 | -0.0026 | -0.0025 |  |
|  | (0.002) | (0.002) | (0.002) |  |
|  | | | |  |
| Nobs | 2429273 | 2429273 | 2429273 |  |
| AIC | 3539062 | 3539192 | 3539254 |  |
| BIC | 3539494 | 3539548 | 3539686 |  |
| LogLik | -1769497 | -1769568 | -1769593 |  |
| Deviance | 3538994 | 3539136 | 3539186 |  |
|  | | | |  |
| *Note:* | ^+^p<0.1; ^*^p<0.05; ^**^p<0.01; ^***^p<0.001 | | | |

**Table 3**

**Staff infections (models 1 - 5 results in the original (log) scale)**

|  | | | | | | | |
| --- | --- | --- | --- | --- | --- | --- | --- |
|  | *Dependent variable:* | | | | | | |
|  |  | | | | | | |
|  | model1: outcome | model2: process | model3: structure (surveys) | model4: structure (provider info) | model5: structure (penalties, staffing) |  |  |
|  | (1) | (2) | (3) | (4) | (5) |  |  |
|  | | | | | |  |  |
| ***conditional model*** |  |  |  |  |  |  |  |
| intercept | 3.522^***^ | 3.3109^***^ | 3.5278^***^ | 3.3505^***^ | 3.5239^***^ |  |  |
|  | (0.0445) | (0.0565) | (0.0565) | (0.0619) | (0.0566) |  |  |
| number_of_residents_with_a_new_positive_covid_19_test_result_out | 0.0207^***^ |  |  |  |  |  |  |
|  | (9e-04) |  |  |  |  |  |  |
| number_of_staff_and_or_personnel_with_a_new_positive_covid_19_test_result_out | 0.5146^***^ |  |  |  |  |  |  |
|  | (0.0018) |  |  |  |  |  |  |
| percentage_of_snf_residents_with_pressure_ulcers_that_are_new_or_worsened_out | -0.0026 |  |  |  |  |  |  |
|  | (0.002) |  |  |  |  |  |  |
| percentage_of_high_risk_long_stay_residents_with_pressure_ulcers_out | 9e-04 |  |  |  |  |  |  |
|  | (0.0023) |  |  |  |  |  |  |
| percentage_of_long_stay_residents_experiencing_one_or_more_falls_with_major_injury_out | 2e-04 |  |  |  |  |  |  |
|  | (0.0022) |  |  |  |  |  |  |
| percentage_of_long_stay_residents_who_have_depressive_symptoms_out | 0 |  |  |  |  |  |  |
|  | (0.0027) |  |  |  |  |  |  |
| percentage_of_long_stay_residents_who_lose_too_much_weight_out | -0.0019 |  |  |  |  |  |  |
|  | (0.0023) |  |  |  |  |  |  |
| percentage_of_long_stay_residents_whose_ability_to_move_independently_worsened_out | 0.0079^**^ |  |  |  |  |  |  |
|  | (0.0029) |  |  |  |  |  |  |
| percentage_of_long_stay_residents_whose_need_for_help_with_daily_activities_has_increased_out | -0.0037 |  |  |  |  |  |  |
|  | (0.0028) |  |  |  |  |  |  |
| percentage_of_long_stay_residents_with_a_urinary_tract_infection_out | -0.0043^+^ |  |  |  |  |  |  |
|  | (0.0023) |  |  |  |  |  |  |
| percentage_of_low_risk_long_stay_residents_who_lose_control_of_their_bowels_or_bladder_out | 0.0021 |  |  |  |  |  |  |
|  | (0.0025) |  |  |  |  |  |  |
| percentage_of_short_stay_residents_who_made_improvements_in_function_out | 0.0013 |  |  |  |  |  |  |
|  | (0.0022) |  |  |  |  |  |  |
| percentage_of_short_stay_residents_who_had_an_outpatient_emergency_department_visit_out | -7e-04 |  |  |  |  |  |  |
|  | (0.0022) |  |  |  |  |  |  |
| percentage_of_short_stay_residents_who_were_rehospitalized_after_a_nursing_home_admission_out | -0.0032 |  |  |  |  |  |  |
|  | (0.0021) |  |  |  |  |  |  |
| residents_weekly_admissions_covid_19_pro |  | 0.0365^***^ |  |  |  |  |  |
|  |  | (0.0012) |  |  |  |  |  |
| residents_hospitalizations_with_confirmed_covid_19_pro |  | 0.0219^***^ |  |  |  |  |  |
|  |  | (0.0012) |  |  |  |  |  |
| residents_hospitalizations_with_confirmed_covid_19_and_up_to_date_with_vaccines_pro |  | 0.0259^***^ |  |  |  |  |  |
|  |  | (0.0045) |  |  |  |  |  |
| percentage_of_current_residents_up_to_date_with_covid_19_vaccines_pro |  | -0.0201^***^ |  |  |  |  |  |
|  |  | (0.0046) |  |  |  |  |  |
| percentage_of_current_healthcare_personnel_up_to_date_with_covid_19_vaccines_pro |  | 0.0083^*^ |  |  |  |  |  |
|  |  | (0.004) |  |  |  |  |  |
| percentage_of_long_stay_residents_assessed_and_appropriately_given_the_pneumococcal_vaccine_pro |  | 4e-04 |  |  |  |  |  |
|  |  | (0.0052) |  |  |  |  |  |
| percentage_of_long_stay_residents_assessed_and_appropriately_given_the_seasonal_influenza_vaccine_pro |  | 1e-04 |  |  |  |  |  |
|  |  | (0.0038) |  |  |  |  |  |
| percentage_of_long_stay_residents_who_received_an_antianxiety_or_hypnotic_medication_pro |  | 0.0075 |  |  |  |  |  |
|  |  | (0.0048) |  |  |  |  |  |
| percentage_of_long_stay_residents_who_received_an_antipsychotic_medication_pro |  | 0.0069 |  |  |  |  |  |
|  |  | (0.0045) |  |  |  |  |  |
| percentage_of_long_stay_residents_who_were_physically_restrained_pro |  | -0.0016 |  |  |  |  |  |
|  |  | (0.0043) |  |  |  |  |  |
| percentage_of_long_stay_residents_with_a_catheter_inserted_and_left_in_their_bladder_pro |  | -0.0072^*^ |  |  |  |  |  |
|  |  | (0.0034) |  |  |  |  |  |
| percentage_of_short_stay_residents_assessed_and_appropriately_given_the_pneumococcal_vaccine_pro |  | 0.0046 |  |  |  |  |  |
|  |  | (0.0063) |  |  |  |  |  |
| percentage_of_short_stay_residents_who_newly_received_an_antipsychotic_medication_pro |  | 5e-04 |  |  |  |  |  |
|  |  | (0.003) |  |  |  |  |  |
| percentage_of_short_stay_residents_who_were_assessed_and_appropriately_given_the_seasonal_influenza_vaccine_pro |  | 0.001 |  |  |  |  |  |
|  |  | (0.0053) |  |  |  |  |  |
| total_health_deficiencies_inspection_cycle_1_str |  |  | 0.0118^**^ |  |  |  |  |
|  |  |  | (0.0045) |  |  |  |  |
| total_health_deficiencies_inspection_cycle_2_str |  |  | 0.0141^***^ |  |  |  |  |
|  |  |  | (0.0041) |  |  |  |  |
| total_health_deficiencies_inspection_cycle_3_str |  |  | 0.0103^**^ |  |  |  |  |
|  |  |  | (0.0038) |  |  |  |  |
| total_fire_deficiencies_inspection_cycle_1_str |  |  | 0.0049 |  |  |  |  |
|  |  |  | (0.0038) |  |  |  |  |
| total_fire_deficiencies_inspection_cycle_2_str |  |  | -0.0055 |  |  |  |  |
|  |  |  | (0.0038) |  |  |  |  |
| total_fire_deficiencies_inspection_cycle_3_str |  |  | 0.0103^**^ |  |  |  |  |
|  |  |  | (0.0037) |  |  |  |  |
| total_weighted_health_survey_score_str |  |  | -0.0185^***^ |  |  |  |  |
|  |  |  | (0.0048) |  |  |  |  |
| percent_of_occupied_beds |  |  |  | -0.0587^***^ |  |  |  |
|  |  |  |  | (0.004) |  |  |  |
| provider_type_str_b11 |  |  |  | 0.0829^**^ |  |  |  |
|  |  |  |  | (0.0254) |  |  |  |
| provider_type_str_b21 |  |  |  | -0.0542^*^ |  |  |  |
|  |  |  |  | (0.0211) |  |  |  |
| provider_resides_in_hospital_str1 |  |  |  | 0.0667^***^ |  |  |  |
|  |  |  |  | (0.0131) |  |  |  |
| days_since_approved_to_provide_medicare_and_medicaid_services_str |  |  |  | 0.0193^***^ |  |  |  |
|  |  |  |  | (0.0048) |  |  |  |
| continuing_care_retirement_community_str1 |  |  |  | -0.0066 |  |  |  |
|  |  |  |  | (0.0082) |  |  |  |
| special_focus_status_str1 |  |  |  | -0.0063^+^ |  |  |  |
|  |  |  |  | (0.0035) |  |  |  |
| abuse_icon_str1 |  |  |  | -5e-04 |  |  |  |
|  |  |  |  | (0.0063) |  |  |  |
| most_recent_health_inspection_more_than_2_years_ago_str1 |  |  |  | -8e-04 |  |  |  |
|  |  |  |  | (0.0041) |  |  |  |
| provider_changed_ownership_in_last_12_months_str1 |  |  |  | 0.0082 |  |  |  |
|  |  |  |  | (0.0097) |  |  |  |
| with_a_resident_and_family_council_str_b11 |  |  |  | 0.0185^***^ |  |  |  |
|  |  |  |  | (0.0052) |  |  |  |
| with_a_resident_and_family_council_str_b21 |  |  |  | 0.0327^**^ |  |  |  |
|  |  |  |  | (0.01) |  |  |  |
| ownership_type_new_str_b11 |  |  |  | 0.015^+^ |  |  |  |
|  |  |  |  | (0.0079) |  |  |  |
| ownership_type_new_str_b21 |  |  |  | -0.0695^***^ |  |  |  |
|  |  |  |  | (0.0136) |  |  |  |
| total_fines_str |  |  |  |  | -0.0106^**^ |  |  |
|  |  |  |  |  | (0.0033) |  |  |
| total_amount_str |  |  |  |  | -0.001 |  |  |
|  |  |  |  |  | (0.0032) |  |  |
| total_penalties_str |  |  |  |  | -0.0089^*^ |  |  |
|  |  |  |  |  | (0.0036) |  |  |
| total_days_str |  |  |  |  | 0.0117^**^ |  |  |
|  |  |  |  |  | (0.0039) |  |  |
| emp_nurse_total_str |  |  |  |  | 0.093^***^ |  |  |
|  |  |  |  |  | (0.0054) |  |  |
| emp_non_nurse_total_str |  |  |  |  | -0.087^***^ |  |  |
|  |  |  |  |  | (0.0048) |  |  |
| ctr_nurse_total_str |  |  |  |  | 0.0753^***^ |  |  |
|  |  |  |  |  | (0.0031) |  |  |
| ctr_non_nurse_total_str |  |  |  |  | -0.0948^***^ |  |  |
|  |  |  |  |  | (0.0041) |  |  |
| number_of_facility_reported_incidents_str |  |  |  |  | -0.001 |  |  |
|  |  |  |  |  | (0.0038) |  |  |
| total_nursing_staff_turnover_str |  |  |  |  | -0.003 |  |  |
|  |  |  |  |  | (0.0031) |  |  |
| registered_nurse_turnover_str |  |  |  |  | -0.0079^**^ |  |  |
|  |  |  |  |  | (0.003) |  |  |
| number_of_administrators_who_have_left_the_nursing_home_str |  |  |  |  | 0.0022 |  |  |
|  |  |  |  |  | (0.0021) |  |  |
|  |  |  |  |  |  |  |  |
|  | | | | | | | |
| ***zero-inflation model*** |  |  |  |  |  |  |  |
| intercept | 1.1979^***^ | 1.1979^***^ | 1.1979^***^ | 1.1979^***^ | 1.1979^***^ |  |  |
|  | (0.0019) | (0.0019) | (0.0019) | (0.0019) | (0.0019) |  |  |
| urban_binary1 | 0.0318^***^ | 0.0318^***^ | 0.0318^***^ | 0.0318^***^ | 0.0318^***^ |  |  |
|  | (0.0019) | (0.0019) | (0.0019) | (0.0019) | (0.0019) |  |  |
| bed_size.L | 0.2173^***^ | 0.2173^***^ | 0.2173^***^ | 0.2173^***^ | 0.2173^***^ |  |  |
|  | (0.0019) | (0.0019) | (0.0019) | (0.0019) | (0.0019) |  |  |
| partially_of_fully_vaccinated_percent | -0.1103^***^ | -0.1103^***^ | -0.1103^***^ | -0.1103^***^ | -0.1103^***^ |  |  |
|  | (0.0015) | (0.0015) | (0.0015) | (0.0015) | (0.0015) |  |  |
| dem_gop1 | -0.0443^***^ | -0.0443^***^ | -0.0443^***^ | -0.0443^***^ | -0.0443^***^ |  |  |
|  | (0.0018) | (0.0018) | (0.0018) | (0.0018) | (0.0018) |  |  |
| median_household_income_dollars_inflation_adjusted_to_data_file_year_acs_2016_2020 | 0.0015 | 0.0015 | 0.0015 | 0.0015 | 0.0015 |  |  |
|  | (0.0016) | (0.0016) | (0.0016) | (0.0016) | (0.0016) |  |  |
|  | | | | | | | |
| Nobs | 2429283 | 2429283 | 2429283 | 2429273 | 2429283 |  |  |
| AIC | 4433971 | 4693110 | 4694582 | 4694154 | 4693527 |  |  |
| BIC | 4434327 | 4693465 | 4694849 | 4694509 | 4693857 |  |  |
| LogLik | -2216958 | -2346527 | -2347270 | -2347049 | -2346737 |  |  |
| Deviance | 4433915 | 4693054 | 4694540 | 4694098 | 4693475 |  |  |
|  | | | | | | | |
| *Note:* | ^+^p<0.1; ^*^p<0.05; ^**^p<0.01; ^***^p<0.001 | | | | | |  |

**Table 4**

**Staff infections (models 6 - 8 results in the original (log) scale)**

|  | | | |  |
| --- | --- | --- | --- | --- |
|  | *Dependent variable:* | | |  |
|  |  | | |  |
|  | Model6: five-star system estimates | Model7: state estimates | Model8: county estimates |  |
|  | (1) | (2) | (3) |  |
|  | | | |  |
| ***conditional model*** |  |  |  |  |
| intercept | 2.9665^***^ | 2.9762^***^ | 2.9565^***^ |  |
|  | (0.0506) | (0.0581) | (0.0679) |  |
| health_inspection_rating.L | 0.0499^***^ |  |  |  |
|  | (0.0056) |  |  |  |
| health_inspection_rating.Q | 0.0187^***^ |  |  |  |
|  | (0.0037) |  |  |  |
| health_inspection_rating.C | 0.0235^***^ |  |  |  |
|  | (0.0032) |  |  |  |
| health_inspection_rating^4 | 1e-04 |  |  |  |
|  | (0.0028) |  |  |  |
| qm_rating.L | 0.0163^**^ |  |  |  |
|  | (0.0055) |  |  |  |
| qm_rating.Q | -0.0057 |  |  |  |
|  | (0.0041) |  |  |  |
| qm_rating.C | -0.0086^**^ |  |  |  |
|  | (0.0033) |  |  |  |
| qm_rating^4 | -0.0059^*^ |  |  |  |
|  | (0.0028) |  |  |  |
| staffing_rating.L | 0.0171^***^ |  |  |  |
|  | (0.0048) |  |  |  |
| staffing_rating.Q | 0.0035 |  |  |  |
|  | (0.0036) |  |  |  |
| staffing_rating.C | -0.0019 |  |  |  |
|  | (0.0029) |  |  |  |
| staffing_rating^4 | -6e-04 |  |  |  |
|  | (0.0024) |  |  |  |
| PC1 | 0.1709^***^ | -0.1527^***^ | -0.1531^***^ |  |
|  | (0.0037) | (0.0032) | (0.0032) |  |
| PC2 | 0.5265^***^ | 0.528^***^ | 0.5279^***^ |  |
|  | (0.0032) | (0.0032) | (0.0032) |  |
| PC3 | -0.0854^***^ | -0.1007^***^ | -0.1007^***^ |  |
|  | (0.0043) | (0.004) | (0.004) |  |
| PC4 | 0.1595^***^ | -0.1623^***^ | -0.1624^***^ |  |
|  | (0.0034) | (0.0034) | (0.0034) |  |
| PC5 | -0.0589^***^ | -0.0473^***^ | -0.0475^***^ |  |
|  | (0.0045) | (0.0045) | (0.0045) |  |
| PC6 | 0.296^***^ | 0.2942^***^ | 0.2942^***^ |  |
|  | (0.0037) | (0.0036) | (0.0036) |  |
| PC7 | 0.0781^***^ | -0.0884^***^ | -0.0886^***^ |  |
|  | (0.0037) | (0.0037) | (0.0037) |  |
| PC8 | -0.2008^***^ | -0.2013^***^ | -0.2014^***^ |  |
|  | (0.0036) | (0.0036) | (0.0036) |  |
| provider_state_b11 |  | -0.0588 |  |  |
|  |  | (0.0511) |  |  |
| provider_state_b21 |  | 0.0314 |  |  |
|  |  | (0.0504) |  |  |
| provider_state_b31 |  | -0.2266^***^ |  |  |
|  |  | (0.0517) |  |  |
| provider_state_b41 |  | -0.0321 |  |  |
|  |  | (0.0516) |  |  |
| provider_state_b51 |  | -0.0656 |  |  |
|  |  | (0.056) |  |  |
| provider_state_b61 |  | 0.0537 |  |  |
|  |  | (0.0585) |  |  |
| provider_state_county_b11 |  |  | 0.0037 |  |
|  |  |  | (0.0092) |  |
| provider_state_county_b21 |  |  | -0.0078 |  |
|  |  |  | (0.0092) |  |
| provider_state_county_b31 |  |  | 0.0058 |  |
|  |  |  | (0.0092) |  |
| provider_state_county_b41 |  |  | 0.0061 |  |
|  |  |  | (0.0092) |  |
| provider_state_county_b51 |  |  | 0.0044 |  |
|  |  |  | (0.0093) |  |
| provider_state_county_b61 |  |  | 0.0081 |  |
|  |  |  | (0.0097) |  |
| provider_state_county_b71 |  |  | 0.0164 |  |
|  |  |  | (0.011) |  |
| provider_state_county_b81 |  |  | 4e-04 |  |
|  |  |  | (0.0147) |  |
| provider_state_county_b91 |  |  | -0.0077 |  |
|  |  |  | (0.0225) |  |
| provider_state_county_b101 |  |  | 0.0242 |  |
|  |  |  | (0.0333) |  |
| provider_state_county_b111 |  |  | 0.0028 |  |
|  |  |  | (0.0492) |  |
| provider_state_county_b121 |  |  | 0.0267 |  |
|  |  |  | (0.0645) |  |
|  |  |  |  |  |
|  | | | |  |
| ***zero-inflation model*** |  |  |  |  |
| intercept | 1.1979^***^ | 1.1979^***^ | 1.1979^***^ |  |
|  | (0.0019) | (0.0019) | (0.0019) |  |
| urban_binary1 | 0.0318^***^ | 0.0318^***^ | 0.0318^***^ |  |
|  | (0.0019) | (0.0019) | (0.0019) |  |
| bed_size.L | 0.2173^***^ | 0.2173^***^ | 0.2173^***^ |  |
|  | (0.0019) | (0.0019) | (0.0019) |  |
| partially_of_fully_vaccinated_percent | -0.1103^***^ | -0.1103^***^ | -0.1103^***^ |  |
|  | (0.0015) | (0.0015) | (0.0015) |  |
| dem_gop1 | -0.0443^***^ | -0.0443^***^ | -0.0443^***^ |  |
|  | (0.0018) | (0.0018) | (0.0018) |  |
| median_household_income_dollars_inflation_adjusted_to_data_file_year_acs_2016_2020 | 0.0014 | 0.0015 | 0.0015 |  |
|  | (0.0016) | (0.0016) | (0.0016) |  |
|  | | | |  |
| Nobs | 2429273 | 2429273 | 2429273 |  |
| AIC | 4644308 | 4644423 | 4644457 |  |
| BIC | 4644740 | 4644778 | 4644889 |  |
| LogLik | -2322120 | -2322183 | -2322194 |  |
| Deviance | 4644240 | 4644367 | 4644389 |  |
|  | | | |  |
| *Note:* | ^+^p<0.1; ^*^p<0.05; ^**^p<0.01; ^***^p<0.001 | | | |

**Table 5**

**Total deaths (models 1 - 5 results in the original (log) scale)**

|  | | | | | |  |
| --- | --- | --- | --- | --- | --- | --- |
|  | *Dependent variable:* | | | | |  |
|  |  | | | | |  |
|  | model1: outcome | model2: process | model3: structure (surveys) | model4: structure (provider info) | model5: structure (penalties, staffing) |  |
|  | (1) | (2) | (3) | (4) | (5) |  |
|  | | | | | |  |
| ***conditional model*** |  |  |  |  |  |  |
| intercept | 3.8944^***^ | 3.3732^***^ | 3.8629^***^ | 3.1252^***^ | 3.8526^***^ |  |
|  | (0.0624) | (0.0634) | (0.0627) | (0.0866) | (0.0628) |  |
| number_of_residents_with_a_new_positive_covid_19_test_result_out | 0.0205^***^ |  |  |  |  |  |
|  | (0.0031) |  |  |  |  |  |
| number_of_staff_and_or_personnel_with_a_new_positive_covid_19_test_result_out | 0.0529^***^ |  |  |  |  |  |
|  | (0.0039) |  |  |  |  |  |
| percentage_of_snf_residents_with_pressure_ulcers_that_are_new_or_worsened_out | 0.0067 |  |  |  |  |  |
|  | (0.0081) |  |  |  |  |  |
| percentage_of_high_risk_long_stay_residents_with_pressure_ulcers_out | -0.0563^***^ |  |  |  |  |  |
|  | (0.01) |  |  |  |  |  |
| percentage_of_long_stay_residents_experiencing_one_or_more_falls_with_major_injury_out | -0.0041 |  |  |  |  |  |
|  | (0.0096) |  |  |  |  |  |
| percentage_of_long_stay_residents_who_have_depressive_symptoms_out | 0.0041 |  |  |  |  |  |
|  | (0.0106) |  |  |  |  |  |
| percentage_of_long_stay_residents_who_lose_too_much_weight_out | 0.0126 |  |  |  |  |  |
|  | (0.0096) |  |  |  |  |  |
| percentage_of_long_stay_residents_whose_ability_to_move_independently_worsened_out | -0.0156 |  |  |  |  |  |
|  | (0.0127) |  |  |  |  |  |
| percentage_of_long_stay_residents_whose_need_for_help_with_daily_activities_has_increased_out | 0.024^*^ |  |  |  |  |  |
|  | (0.0116) |  |  |  |  |  |
| percentage_of_long_stay_residents_with_a_urinary_tract_infection_out | -0.0301^**^ |  |  |  |  |  |
|  | (0.0093) |  |  |  |  |  |
| percentage_of_low_risk_long_stay_residents_who_lose_control_of_their_bowels_or_bladder_out | -0.0023 |  |  |  |  |  |
|  | (0.0095) |  |  |  |  |  |
| percentage_of_short_stay_residents_who_made_improvements_in_function_out | 0.0139 |  |  |  |  |  |
|  | (0.0087) |  |  |  |  |  |
| percentage_of_short_stay_residents_who_had_an_outpatient_emergency_department_visit_out | 0.0033 |  |  |  |  |  |
|  | (0.0093) |  |  |  |  |  |
| percentage_of_short_stay_residents_who_were_rehospitalized_after_a_nursing_home_admission_out | -0.0045 |  |  |  |  |  |
|  | (0.0091) |  |  |  |  |  |
| residents_weekly_admissions_covid_19_pro |  | 0.048^***^ |  |  |  |  |
|  |  | (0.0021) |  |  |  |  |
| residents_hospitalizations_with_confirmed_covid_19_pro |  | 0.005 |  |  |  |  |
|  |  | (0.0044) |  |  |  |  |
| residents_hospitalizations_with_confirmed_covid_19_and_up_to_date_with_vaccines_pro |  | 0.0541^**^ |  |  |  |  |
|  |  | (0.0167) |  |  |  |  |
| percentage_of_current_residents_up_to_date_with_covid_19_vaccines_pro |  | -0.1032^**^ |  |  |  |  |
|  |  | (0.0326) |  |  |  |  |
| percentage_of_current_healthcare_personnel_up_to_date_with_covid_19_vaccines_pro |  | -5e-04 |  |  |  |  |
|  |  | (0.028) |  |  |  |  |
| percentage_of_long_stay_residents_assessed_and_appropriately_given_the_pneumococcal_vaccine_pro |  | -0.0232^+^ |  |  |  |  |
|  |  | (0.0126) |  |  |  |  |
| percentage_of_long_stay_residents_assessed_and_appropriately_given_the_seasonal_influenza_vaccine_pro |  | 0.0363^***^ |  |  |  |  |
|  |  | (0.0098) |  |  |  |  |
| percentage_of_long_stay_residents_who_received_an_antianxiety_or_hypnotic_medication_pro |  | 0.0123 |  |  |  |  |
|  |  | (0.0107) |  |  |  |  |
| percentage_of_long_stay_residents_who_received_an_antipsychotic_medication_pro |  | 0.02^+^ |  |  |  |  |
|  |  | (0.0106) |  |  |  |  |
| percentage_of_long_stay_residents_who_were_physically_restrained_pro |  | 0.0194^+^ |  |  |  |  |
|  |  | (0.0113) |  |  |  |  |
| percentage_of_long_stay_residents_with_a_catheter_inserted_and_left_in_their_bladder_pro |  | -0.0245^**^ |  |  |  |  |
|  |  | (0.0094) |  |  |  |  |
| percentage_of_short_stay_residents_assessed_and_appropriately_given_the_pneumococcal_vaccine_pro |  | 0.0251 |  |  |  |  |
|  |  | (0.0164) |  |  |  |  |
| percentage_of_short_stay_residents_who_newly_received_an_antipsychotic_medication_pro |  | 0.0143 |  |  |  |  |
|  |  | (0.0088) |  |  |  |  |
| percentage_of_short_stay_residents_who_were_assessed_and_appropriately_given_the_seasonal_influenza_vaccine_pro |  | -0.0324^*^ |  |  |  |  |
|  |  | (0.0144) |  |  |  |  |
| total_health_deficiencies_inspection_cycle_1_str |  |  | 0.0198^+^ |  |  |  |
|  |  |  | (0.0116) |  |  |  |
| total_health_deficiencies_inspection_cycle_2_str |  |  | 0.011 |  |  |  |
|  |  |  | (0.0107) |  |  |  |
| total_health_deficiencies_inspection_cycle_3_str |  |  | 0.0166 |  |  |  |
|  |  |  | (0.0102) |  |  |  |
| total_fire_deficiencies_inspection_cycle_1_str |  |  | 0.0022 |  |  |  |
|  |  |  | (0.0101) |  |  |  |
| total_fire_deficiencies_inspection_cycle_2_str |  |  | -0.0078 |  |  |  |
|  |  |  | (0.0102) |  |  |  |
| total_fire_deficiencies_inspection_cycle_3_str |  |  | 0.0196^+^ |  |  |  |
|  |  |  | (0.0106) |  |  |  |
| total_weighted_health_survey_score_str |  |  | -0.0139 |  |  |  |
|  |  |  | (0.0129) |  |  |  |
| percent_of_occupied_beds |  |  |  | -0.2545^***^ |  |  |
|  |  |  |  | (0.0095) |  |  |
| provider_type_str_b11 |  |  |  | 0.2508^***^ |  |  |
|  |  |  |  | (0.0577) |  |  |
| provider_type_str_b21 |  |  |  | -0.0802^+^ |  |  |
|  |  |  |  | (0.0477) |  |  |
| provider_resides_in_hospital_str1 |  |  |  | 0.1292^***^ |  |  |
|  |  |  |  | (0.0316) |  |  |
| days_since_approved_to_provide_medicare_and_medicaid_services_str |  |  |  | 0.0424^***^ |  |  |
|  |  |  |  | (0.0095) |  |  |
| continuing_care_retirement_community_str1 |  |  |  | 0.059^***^ |  |  |
|  |  |  |  | (0.016) |  |  |
| special_focus_status_str1 |  |  |  | -0.0092 |  |  |
|  |  |  |  | (0.0087) |  |  |
| abuse_icon_str1 |  |  |  | -0.0221 |  |  |
|  |  |  |  | (0.0176) |  |  |
| most_recent_health_inspection_more_than_2_years_ago_str1 |  |  |  | 0.0306 |  |  |
|  |  |  |  | (0.0194) |  |  |
| provider_changed_ownership_in_last_12_months_str1 |  |  |  | 0.028 |  |  |
|  |  |  |  | (0.0272) |  |  |
| with_a_resident_and_family_council_str_b11 |  |  |  | 0.0548^***^ |  |  |
|  |  |  |  | (0.011) |  |  |
| with_a_resident_and_family_council_str_b21 |  |  |  | 0.1571^***^ |  |  |
|  |  |  |  | (0.0275) |  |  |
| ownership_type_new_str_b11 |  |  |  | 0.0365^**^ |  |  |
|  |  |  |  | (0.0115) |  |  |
| ownership_type_new_str_b21 |  |  |  | -0.0285 |  |  |
|  |  |  |  | (0.0194) |  |  |
| total_fines_str |  |  |  |  | 0.0097 |  |
|  |  |  |  |  | (0.0162) |  |
| total_amount_str |  |  |  |  | 0.0016 |  |
|  |  |  |  |  | (0.0093) |  |
| total_penalties_str |  |  |  |  | -0.0023 |  |
|  |  |  |  |  | (0.01) |  |
| total_days_str |  |  |  |  | 0.004 |  |
|  |  |  |  |  | (0.011) |  |
| emp_nurse_total_str |  |  |  |  | 0.0643^***^ |  |
|  |  |  |  |  | (0.011) |  |
| emp_non_nurse_total_str |  |  |  |  | -0.045^***^ |  |
|  |  |  |  |  | (0.0104) |  |
| ctr_nurse_total_str |  |  |  |  | 0.1016^***^ |  |
|  |  |  |  |  | (0.0078) |  |
| ctr_non_nurse_total_str |  |  |  |  | -0.0444^***^ |  |
|  |  |  |  |  | (0.0093) |  |
| number_of_facility_reported_incidents_str |  |  |  |  | 0.018^+^ |  |
|  |  |  |  |  | (0.0094) |  |
| total_nursing_staff_turnover_str |  |  |  |  | 0.0136 |  |
|  |  |  |  |  | (0.0091) |  |
| registered_nurse_turnover_str |  |  |  |  | 0.0147^+^ |  |
|  |  |  |  |  | (0.0087) |  |
| number_of_administrators_who_have_left_the_nursing_home_str |  |  |  |  | 0.0022 |  |
|  |  |  |  |  | (0.0061) |  |
|  |  |  |  |  |  |  |
|  |  |  |  |  |  |  |
|  |  |  |  |  |  |  |
|  |  |  |  |  |  |  |
|  |  |  |  |  |  |  |
|  |  |  |  |  |  |  |
|  |  |  |  |  |  |  |
|  |  |  |  |  |  |  |
|  |  |  |  |  |  |  |
|  |  |  |  |  |  |  |
|  |  |  |  |  |  |  |
|  |  |  |  |  |  |  |
|  |  |  |  |  |  |  |
|  |  |  |  |  |  |  |
|  |  |  |  |  |  |  |
|  |  |  |  |  |  |  |
|  |  |  |  |  |  |  |
|  |  |  |  |  |  |  |
|  |  |  |  |  |  |  |
|  |  |  |  |  |  |  |
|  |  |  |  |  |  |  |
|  |  |  |  |  |  |  |
|  |  |  |  |  |  |  |
|  |  |  |  |  |  |  |
|  |  |  |  |  |  |  |
|  |  |  |  |  |  |  |
|  |  |  |  |  |  |  |
|  |  |  |  |  |  |  |
|  |  |  |  |  |  |  |
|  |  |  |  |  |  |  |
|  |  |  |  |  |  |  |
|  |  |  |  |  |  |  |
|  |  |  |  |  |  |  |
|  |  |  |  |  |  |  |
|  |  |  |  |  |  |  |
|  |  |  |  |  |  |  |
|  |  |  |  |  |  |  |
|  |  |  |  |  |  |  |
|  |  |  |  |  |  |  |
|  |  |  |  |  |  |  |
|  |  |  |  |  |  |  |
|  |  |  |  |  |  |  |
|  |  |  |  |  |  |  |
|  |  |  |  |  |  |  |
|  |  |  |  |  |  |  |
|  |  |  |  |  |  |  |
|  |  |  |  |  |  |  |
|  |  |  |  |  |  |  |
|  |  |  |  |  |  |  |
|  |  |  |  |  |  |  |
|  |  |  |  |  |  |  |
|  | | | | | |  |
| ***zero-inflation model*** |  |  |  |  |  |  |
| intercept | 3.4912^***^ | 3.4912^***^ | 3.4912^***^ | 3.4912^***^ | 3.4912^***^ |  |
|  | (0.0038) | (0.0038) | (0.0038) | (0.0038) | (0.0038) |  |
| lagged_weekly_resident_confirmed_covid_19_cases_per_1_000_residents | -0.5544^***^ | -0.5544^***^ | -0.5544^***^ | -0.5544^***^ | -0.5544^***^ |  |
|  | (0.0022) | (0.0022) | (0.0022) | (0.0022) | (0.0022) |  |
| lagged_county_confirmed_cases_usafacts_new | -0.0869^***^ | -0.0869^***^ | -0.0869^***^ | -0.0869^***^ | -0.0869^***^ |  |
|  | (0.004) | (0.004) | (0.004) | (0.004) | (0.004) |  |
|  | | | | | |  |
| Nobs | 2429273 | 2429273 | 2429273 | 2429273 | 2429273 |  |
| AIC | 851851 | 851743 | 852422 | 851459 | 852226 |  |
| BIC | 852168 | 852060 | 852651 | 851777 | 852518 |  |
| LogLik | -425900 | -425846 | -426193 | -425705 | -426090 |  |
| Deviance | 851801 | 851693 | 852386 | 851409 | 852180 |  |
|  | | | | | |  |
| *Note:* | ^+^p<0.1; ^*^p<0.05; ^**^p<0.01; ^***^p<0.001 | | | | | |

**Table 6**

**Total deaths (model 6 - 8 results in the original (log) scale)**

|  | | | |  |
| --- | --- | --- | --- | --- |
|  | *Dependent variable:* | | |  |
|  |  | | |  |
|  | model6: five-star system estimates | model7: state estimates | model8: county estimates |  |
|  | (1) | (2) | (3) |  |
|  | | | |  |
| ***conditional model*** |  |  |  |  |
| intercept | 3.4501^***^ | 3.4465^***^ | 3.4732^***^ |  |
|  | (0.0597) | (0.072) | (0.0842) |  |
| health_inspection_rating.L | 0.0206 |  |  |  |
|  | (0.0145) |  |  |  |
| health_inspection_rating.Q | 0.004 |  |  |  |
|  | (0.0096) |  |  |  |
| health_inspection_rating.C | 0.0113 |  |  |  |
|  | (0.0086) |  |  |  |
| health_inspection_rating^4 | -0.0156^*^ |  |  |  |
|  | (0.0079) |  |  |  |
| qm_rating.L | 0.0088 |  |  |  |
|  | (0.0144) |  |  |  |
| qm_rating.Q | -0.0238^*^ |  |  |  |
|  | (0.0116) |  |  |  |
| qm_rating.C | 0.0196^*^ |  |  |  |
|  | (0.0098) |  |  |  |
| qm_rating^4 | -0.0111 |  |  |  |
|  | (0.0084) |  |  |  |
| staffing_rating.L | -0.1175^***^ |  |  |  |
|  | (0.0136) |  |  |  |
| staffing_rating.Q | -0.045^***^ |  |  |  |
|  | (0.0105) |  |  |  |
| staffing_rating.C | -3e-04 |  |  |  |
|  | (0.0085) |  |  |  |
| staffing_rating^4 | -0.0137^+^ |  |  |  |
|  | (0.0071) |  |  |  |
| PC1 | 0.0362^***^ | -0.046^***^ | -0.0474^***^ |  |
|  | (0.0083) | (0.0063) | (0.0063) |  |
| PC2 | 0.0239^***^ | 0.0279^***^ | 0.0276^***^ |  |
|  | (0.0062) | (0.006) | (0.006) |  |
| PC3 | -0.0172^+^ | -0.015^+^ | -0.014^+^ |  |
|  | (0.009) | (0.0083) | (0.0083) |  |
| PC4 | -0.0222^**^ | 0.0248^***^ | 0.0248^***^ |  |
|  | (0.0075) | (0.0075) | (0.0075) |  |
| PC5 | 0.026^**^ | 0.0316^***^ | 0.0313^***^ |  |
|  | (0.0092) | (0.0091) | (0.0091) |  |
| PC6 | 0.2085^***^ | 0.1863^***^ | 0.1868^***^ |  |
|  | (0.0089) | (0.0087) | (0.0087) |  |
| PC7 | 0.2498^***^ | -0.2608^***^ | -0.2609^***^ |  |
|  | (0.0094) | (0.0093) | (0.0093) |  |
| PC8 | -0.0753^***^ | -0.0706^***^ | -0.0711^***^ |  |
|  | (0.0085) | (0.0085) | (0.0085) |  |
| provider_state_b11 |  | 0.0429 |  |  |
|  |  | (0.0597) |  |  |
| provider_state_b21 |  | -0.04 |  |  |
|  |  | (0.0585) |  |  |
| provider_state_b31 |  | -0.2035^***^ |  |  |
|  |  | (0.0616) |  |  |
| provider_state_b41 |  | 0.1091^+^ |  |  |
|  |  | (0.0597) |  |  |
| provider_state_b51 |  | -0.084 |  |  |
|  |  | (0.0655) |  |  |
| provider_state_b61 |  | -0.0592 |  |  |
|  |  | (0.0713) |  |  |
| provider_state_county_b11 |  |  | 0.0207^+^ |  |
|  |  |  | (0.0108) |  |
| provider_state_county_b21 |  |  | 0.0011 |  |
|  |  |  | (0.0108) |  |
| provider_state_county_b31 |  |  | 0.0053 |  |
|  |  |  | (0.0108) |  |
| provider_state_county_b41 |  |  | 0.0019 |  |
|  |  |  | (0.0109) |  |
| provider_state_county_b51 |  |  | 0.0079 |  |
|  |  |  | (0.011) |  |
| provider_state_county_b61 |  |  | 0.0036 |  |
|  |  |  | (0.0116) |  |
| provider_state_county_b71 |  |  | -0.0048 |  |
|  |  |  | (0.0131) |  |
| provider_state_county_b81 |  |  | 0.0156 |  |
|  |  |  | (0.0177) |  |
| provider_state_county_b91 |  |  | 0.0173 |  |
|  |  |  | (0.0278) |  |
| provider_state_county_b101 |  |  | -0.0071 |  |
|  |  |  | (0.0405) |  |
| provider_state_county_b111 |  |  | -0.1014^+^ |  |
|  |  |  | (0.0569) |  |
| provider_state_county_b121 |  |  | 0.0014 |  |
|  |  |  | (0.0782) |  |
|  |  |  |  |  |
|  | | | |  |
| ***zero-inflation model*** |  |  |  |  |
| intercept | 3.4912^***^ | 3.4912^***^ | 3.4912^***^ |  |
|  | (0.0038) | (0.0038) | (0.0038) |  |
| lagged_weekly_resident_confirmed_covid_19_cases_per_1_000_residents | -0.5544^***^ | -0.5544^***^ | -0.5544^***^ |  |
|  | (0.0022) | (0.0022) | (0.0022) |  |
| lagged_county_confirmed_cases_usafacts_new | -0.0869^***^ | -0.0869^***^ | -0.0869^***^ |  |
|  | (0.004) | (0.004) | (0.004) |  |
|  | | | |  |
| Nobs | 2429273 | 2429273 | 2429273 |  |
| AIC | 850900 | 850976 | 850998 |  |
| BIC | 851294 | 851294 | 851392 |  |
| LogLik | -425419 | -425463 | -425468 |  |
| Deviance | 850838 | 850926 | 850936 |  |
|  | | | |  |
| *Note:* | ^+^p<0.1; ^*^p<0.05; ^**^p<0.01; ^***^p<0.001 | | | |
