# Supplementary material for: Demystifying Quality Metrics and Unveiling the True Measure of Quality of Care in Nursing Homes: Mixed Effects Analysis
Source: JMIR Hum Factors. 2026 Jan 29;13:e72770. doi: 10.2196/72770 (PMC12854662; doi:10.2196/72770)
Supplement: Multimedia Appendix 2 [file humanfactors-v13-e72770-s002.docx]

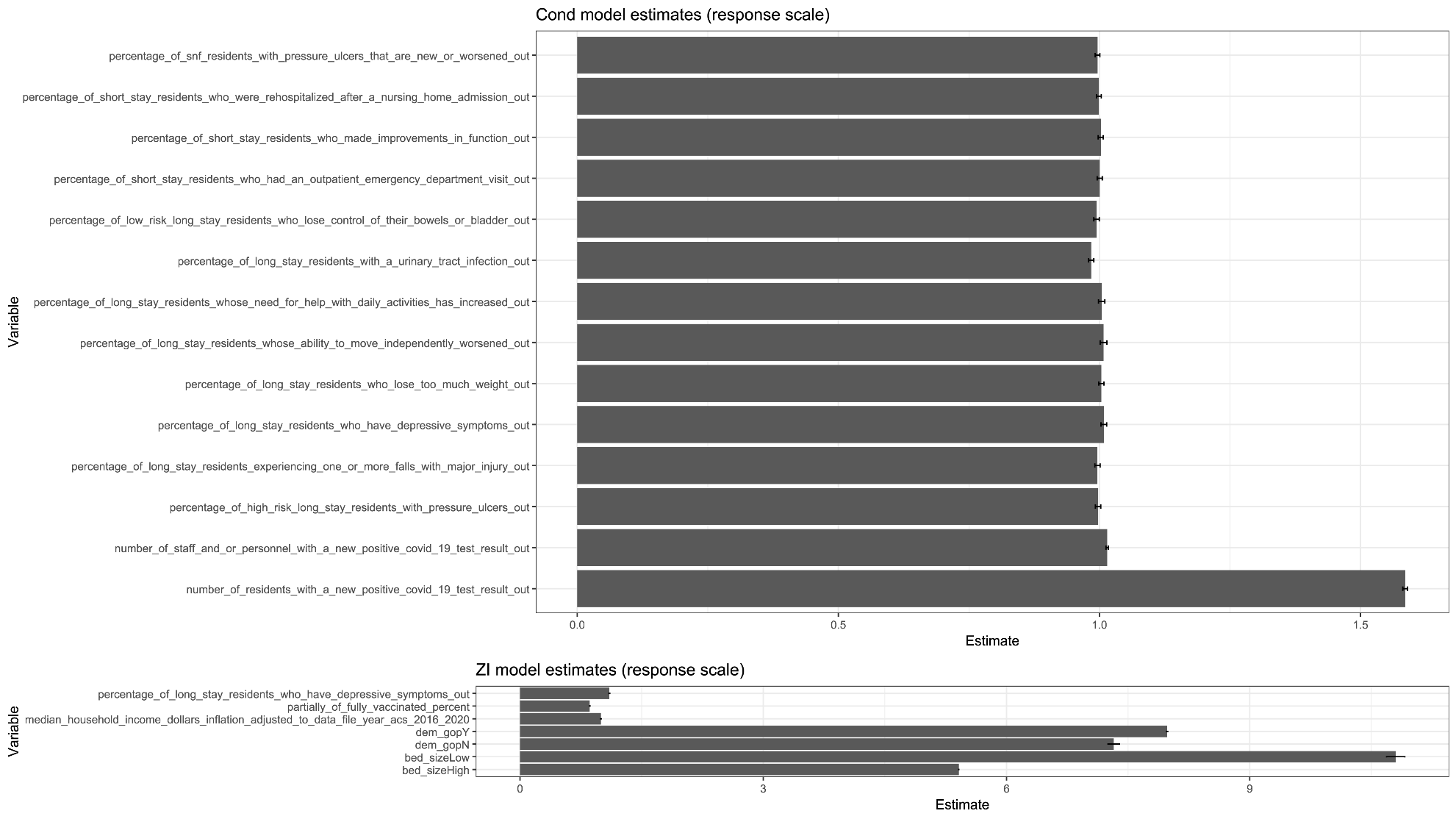


**Figure 7**

Resident infections (model 1 estimates in the response (exponent) scale)


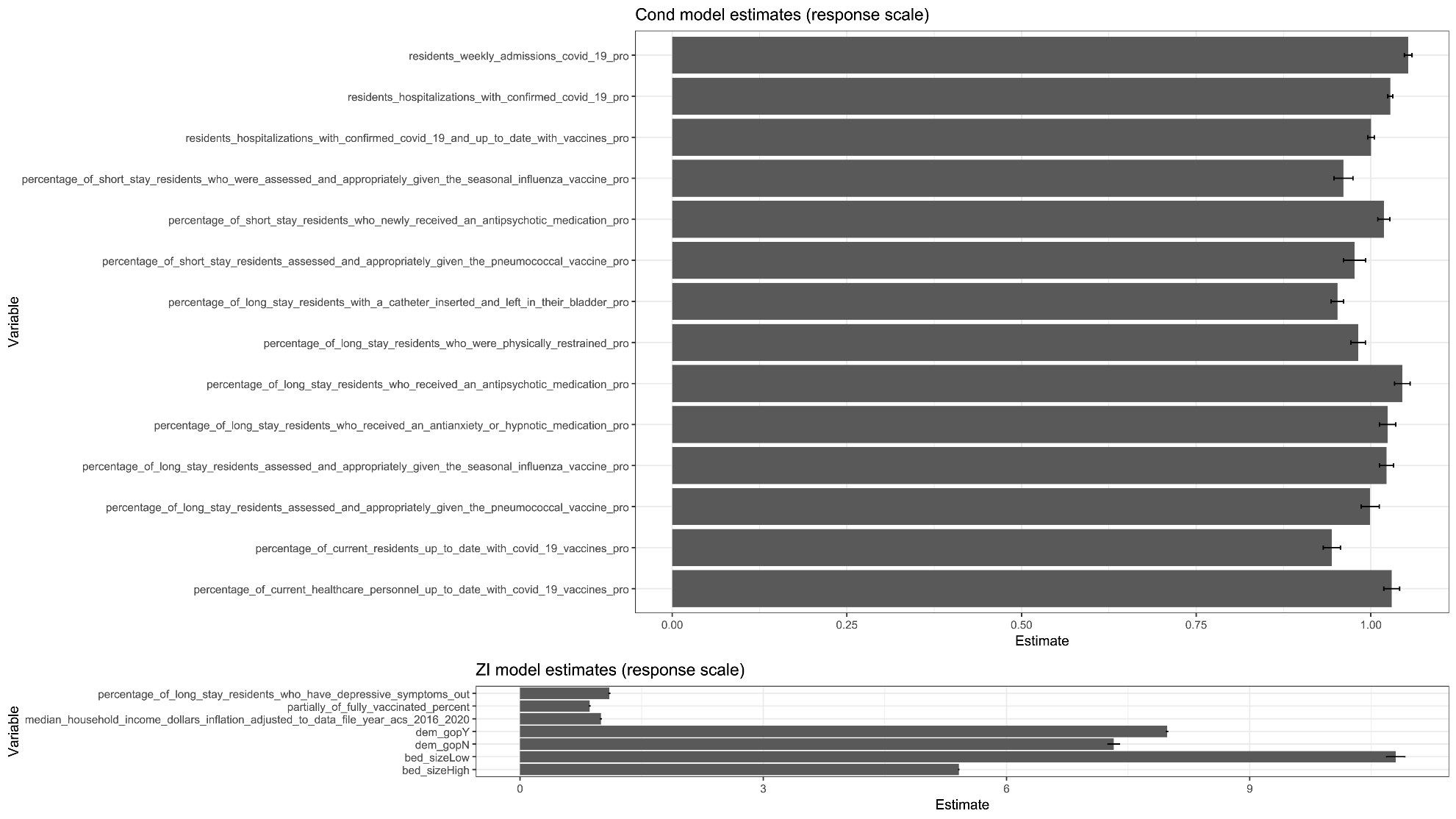


**Figure 8**

Resident infections (model 2 estimates in the response (exponent) scale)


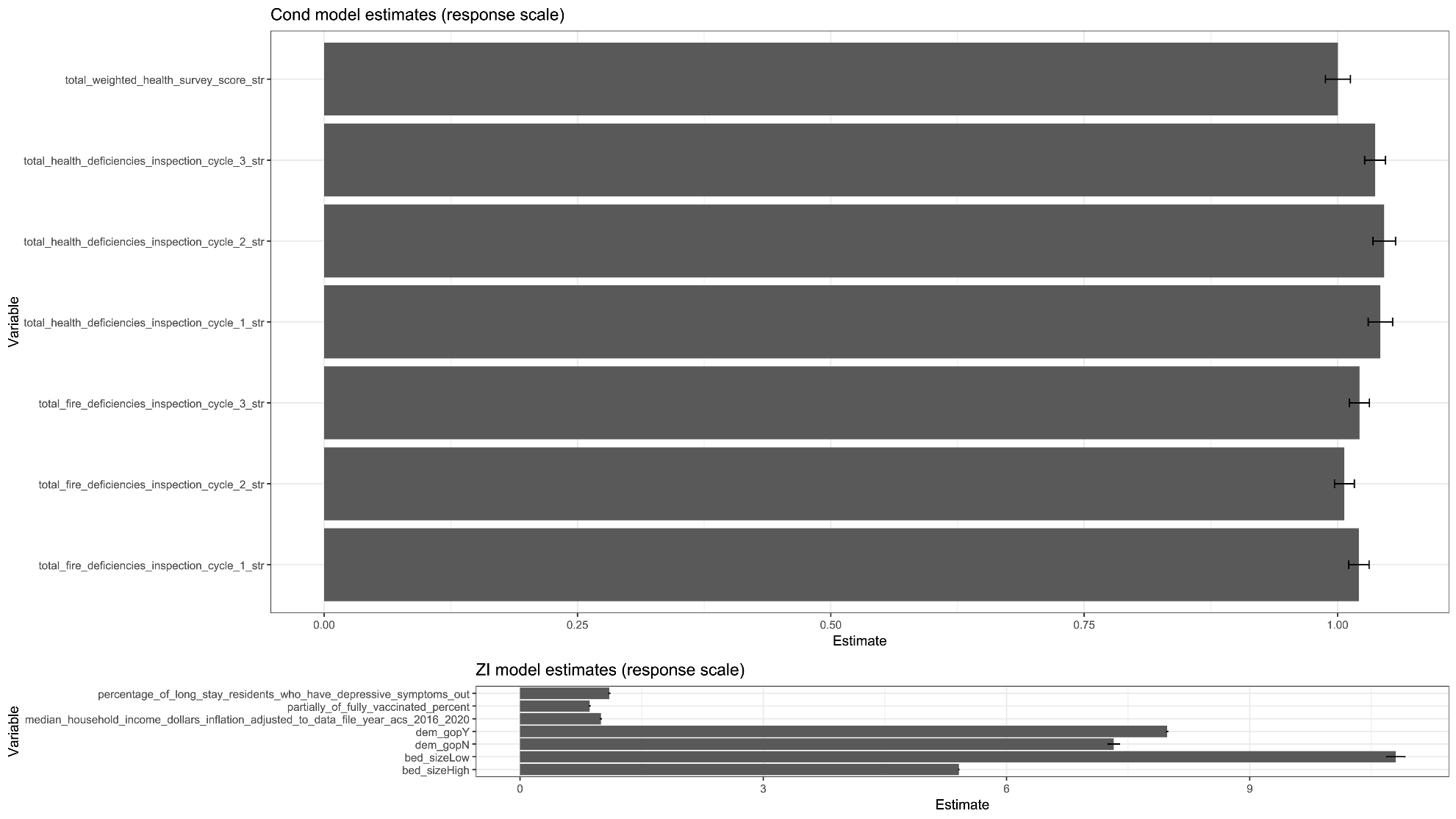


**Figure 9**

Resident infections (model 3 estimates in the response (exponent) scale)


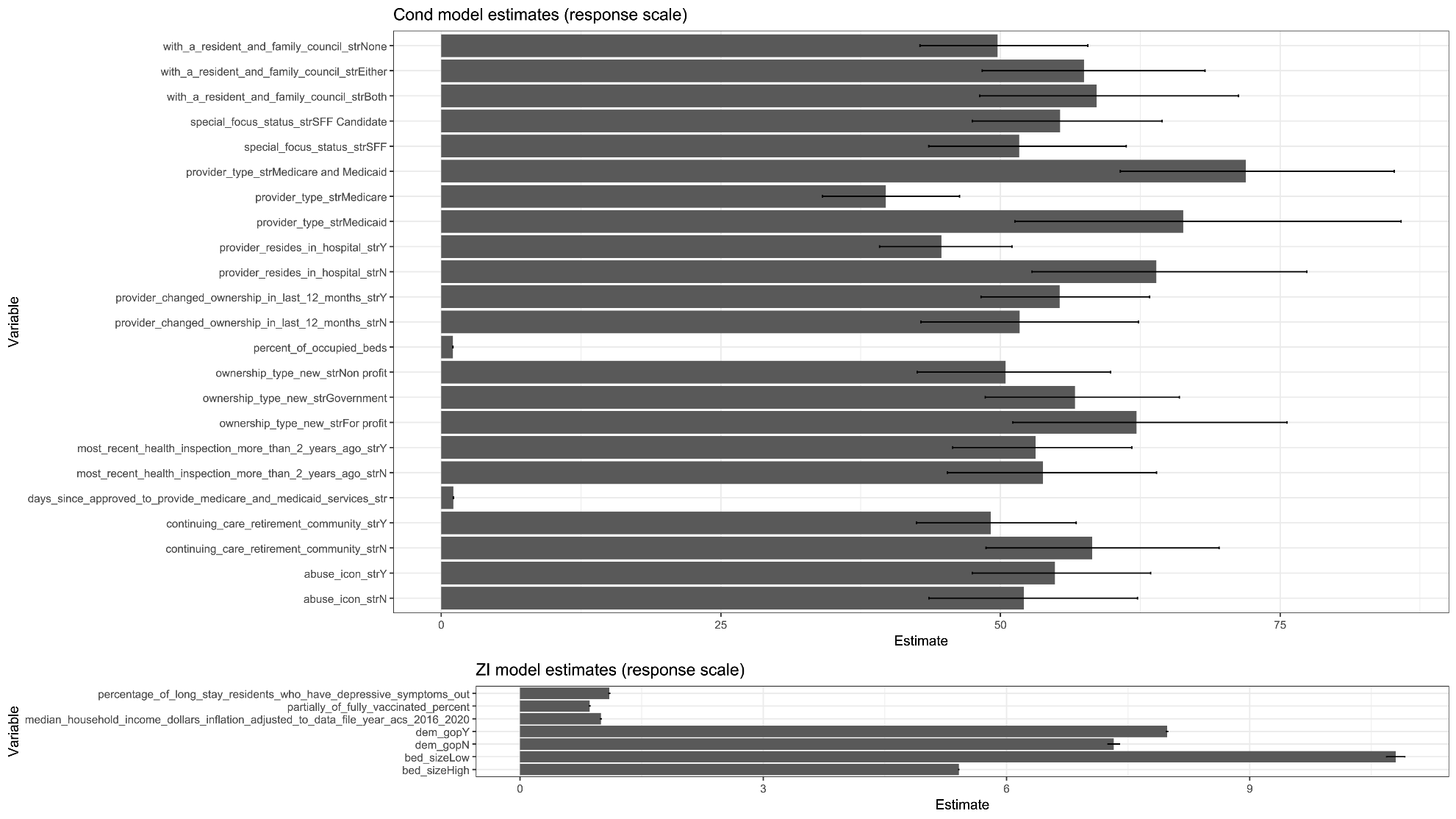


**Figure 10**

Resident infections (model 4 estimates in the response (exponent) scale)


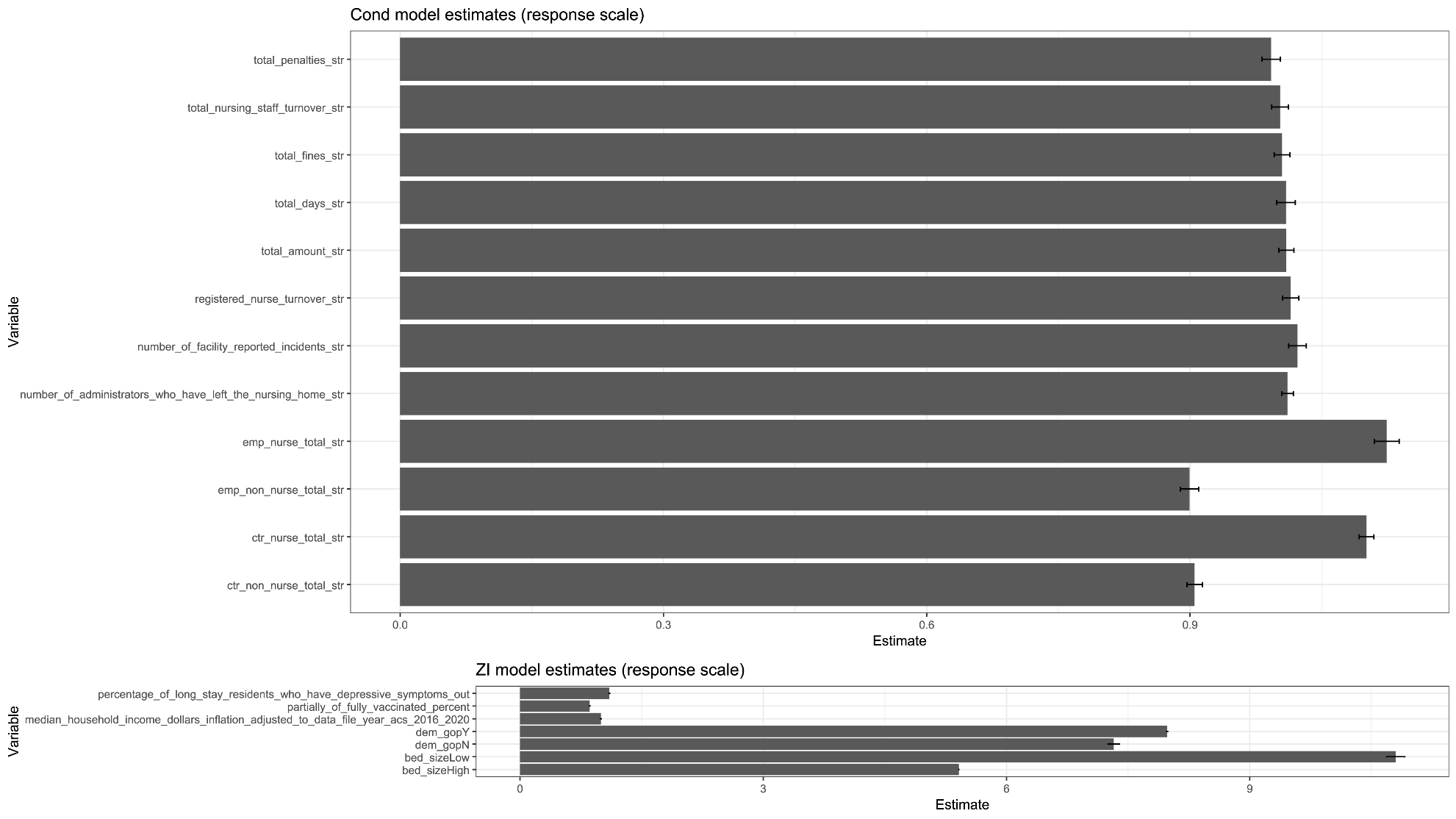


**Figure 11**

Resident infections (model 5 estimates in the response (exponent) scale)


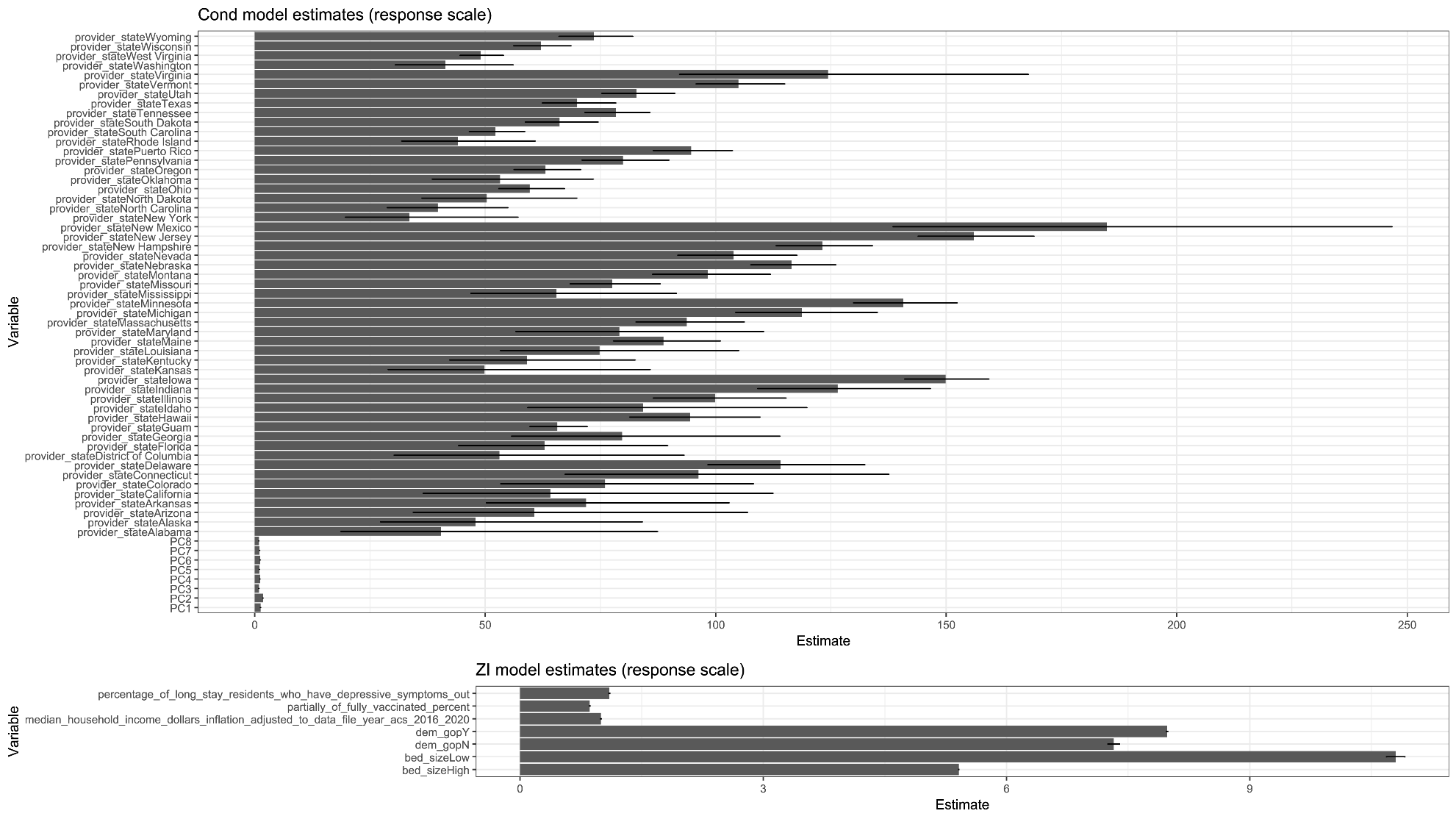


**Figure 12**

Resident infections (model 6 estimates in the response (exponent) scale)


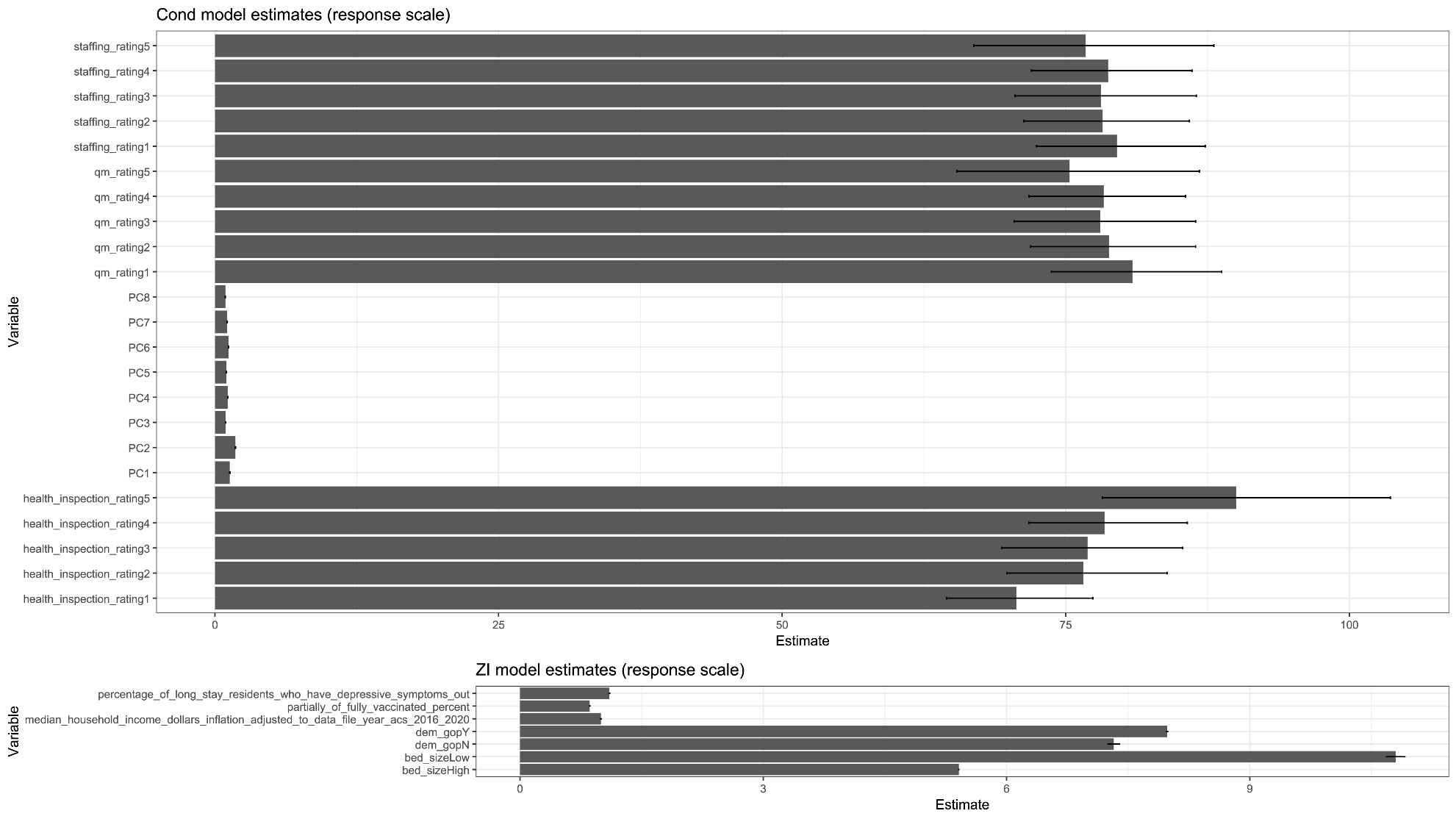


**Figure 13**

Resident infections (model 7 estimates in the response (exponent) scale)


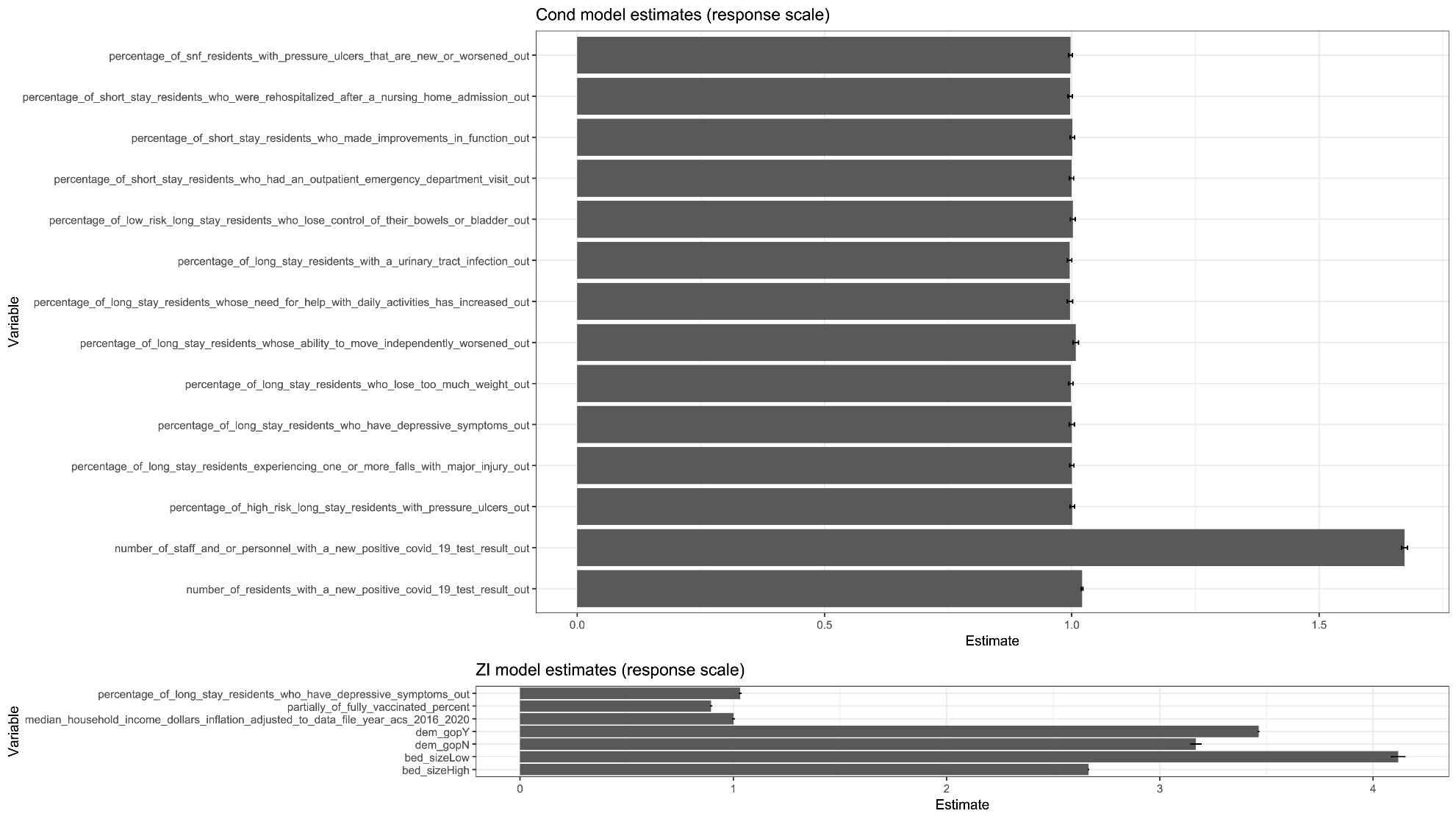


**Figure 14**

Staff infections (model 1 estimates in the response (exponent) scale)


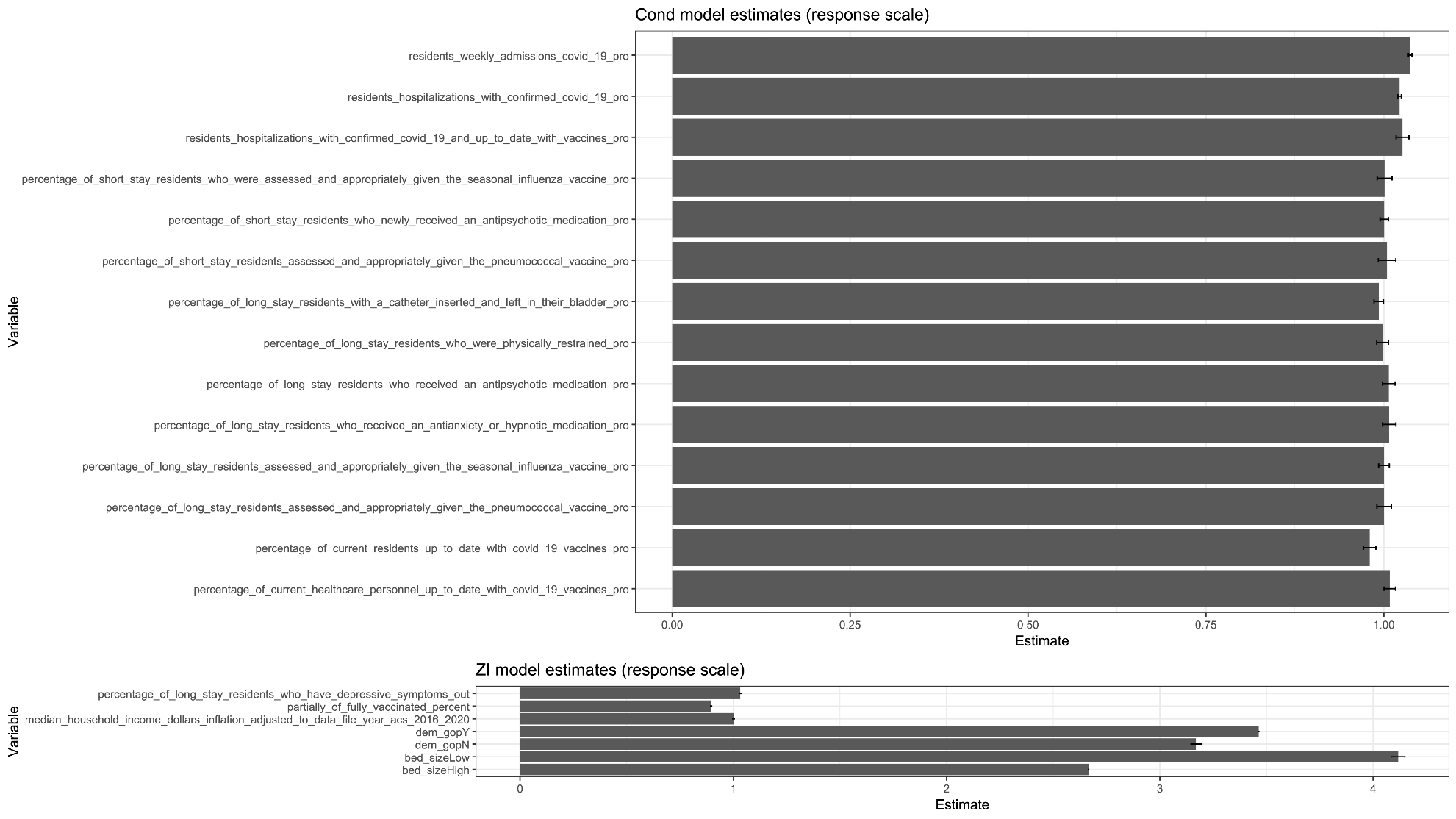


**Figure 15**

Staff infections (model 2 estimates in the response (exponent) scale)


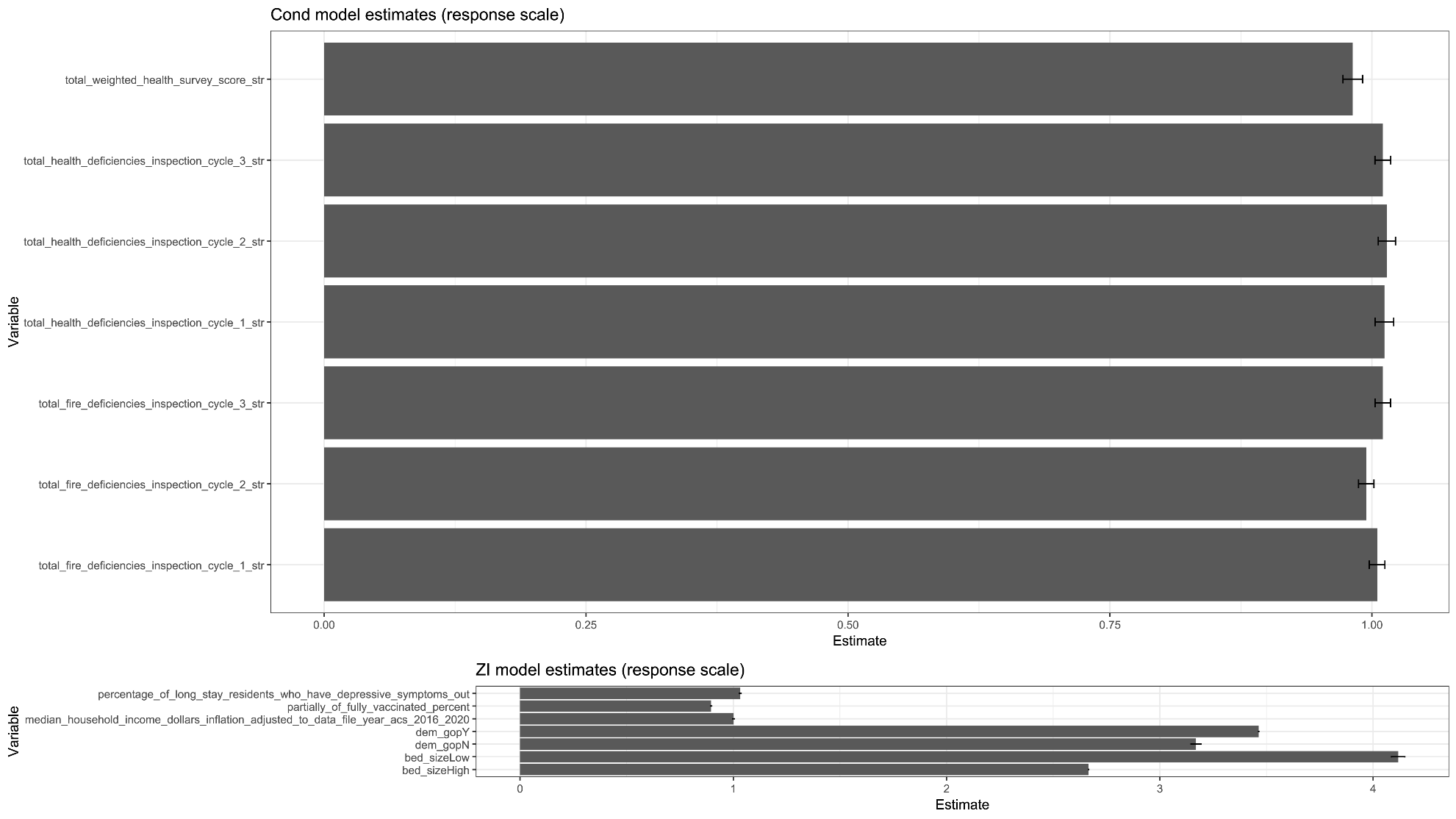


**Figure 16**

Staff infections (model 3 estimates in the response (exponent) scale)


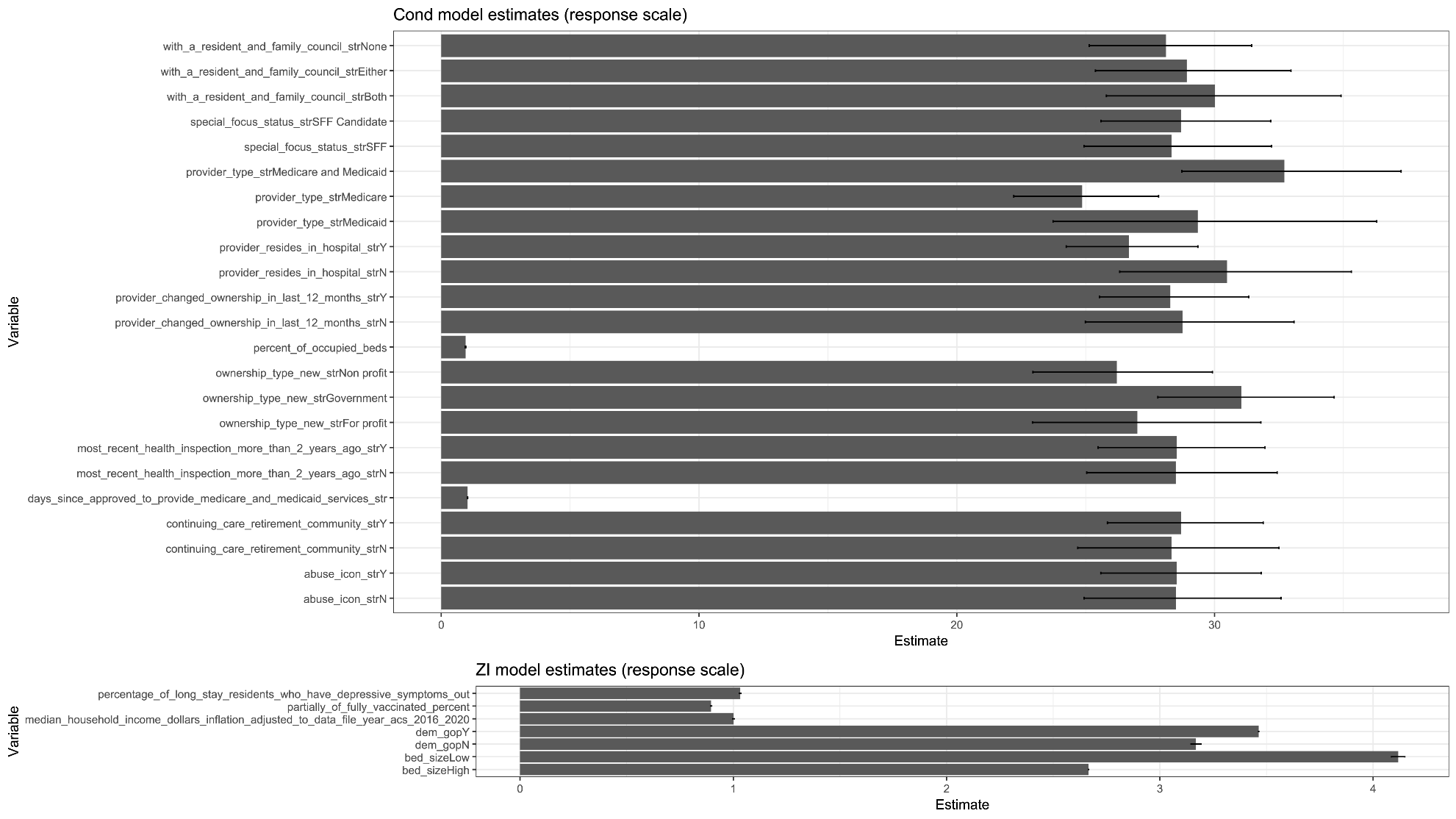


**Figure 17**

Staff infections (model 4 estimates in the response (exponent) scale)


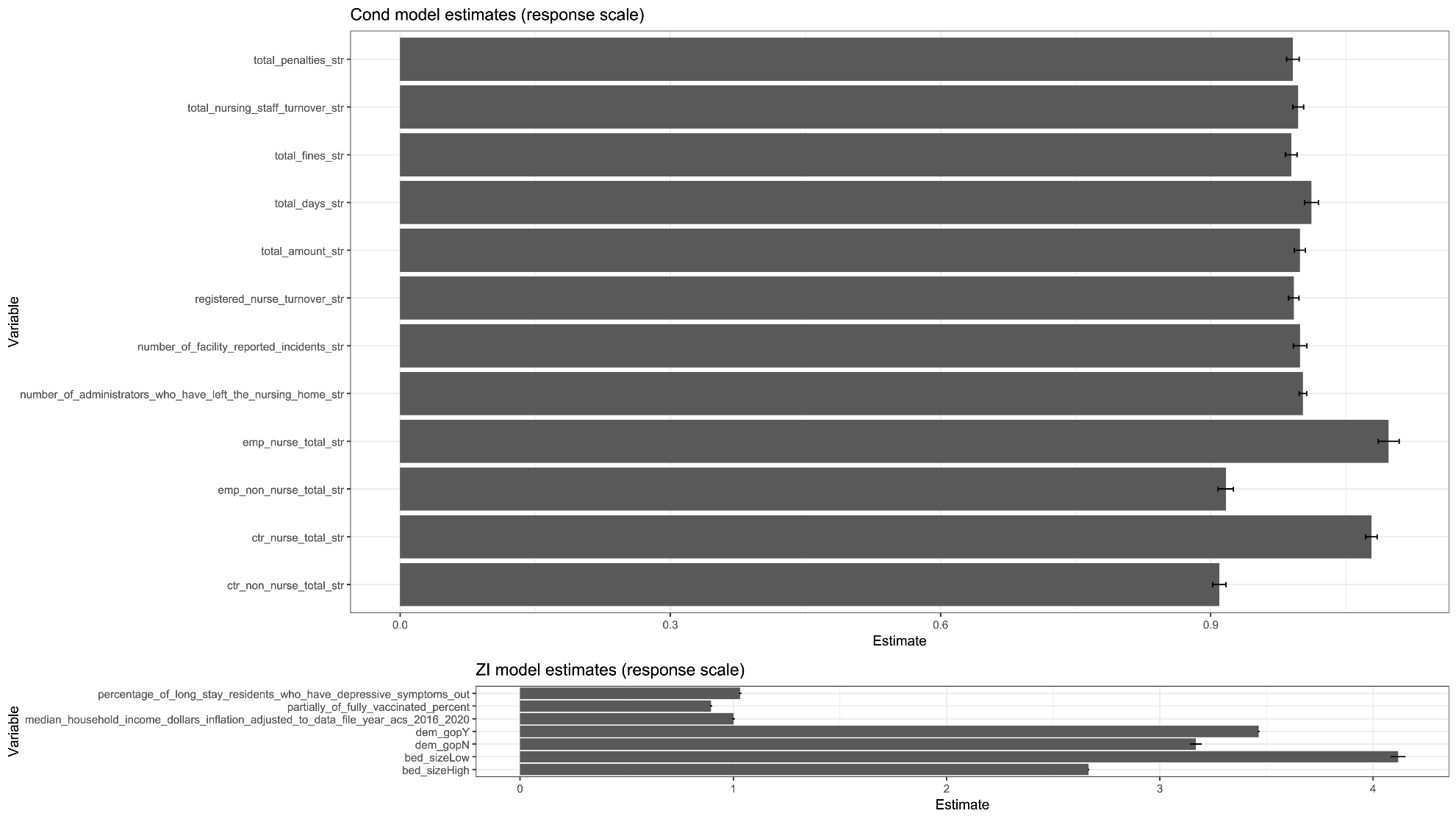


**Figure 18**

Staff infections (model 5 estimates in the response (exponent) scale)


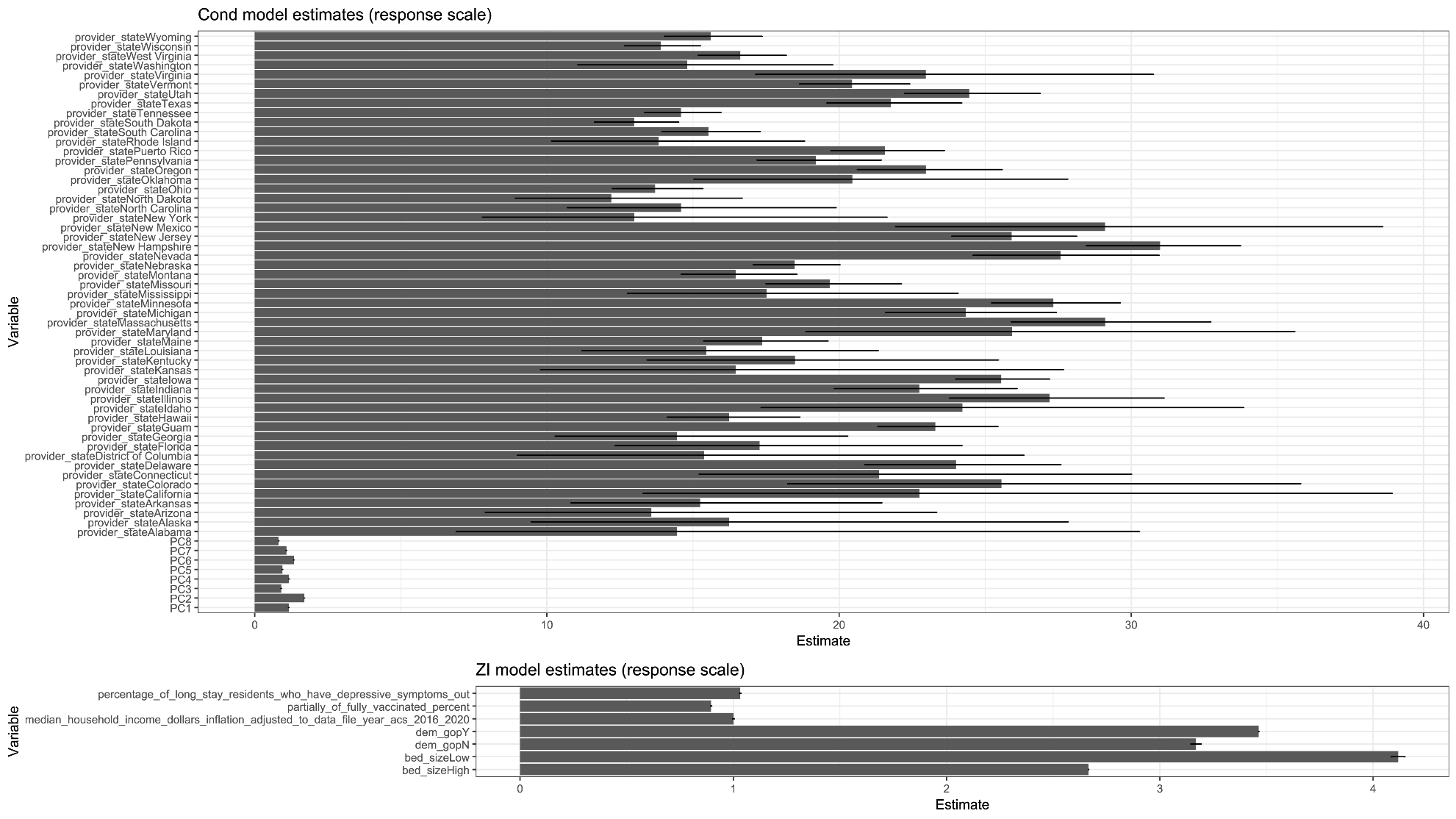


**Figure 19**

Staff infections (model 6 estimates in the response (exponent) scale)


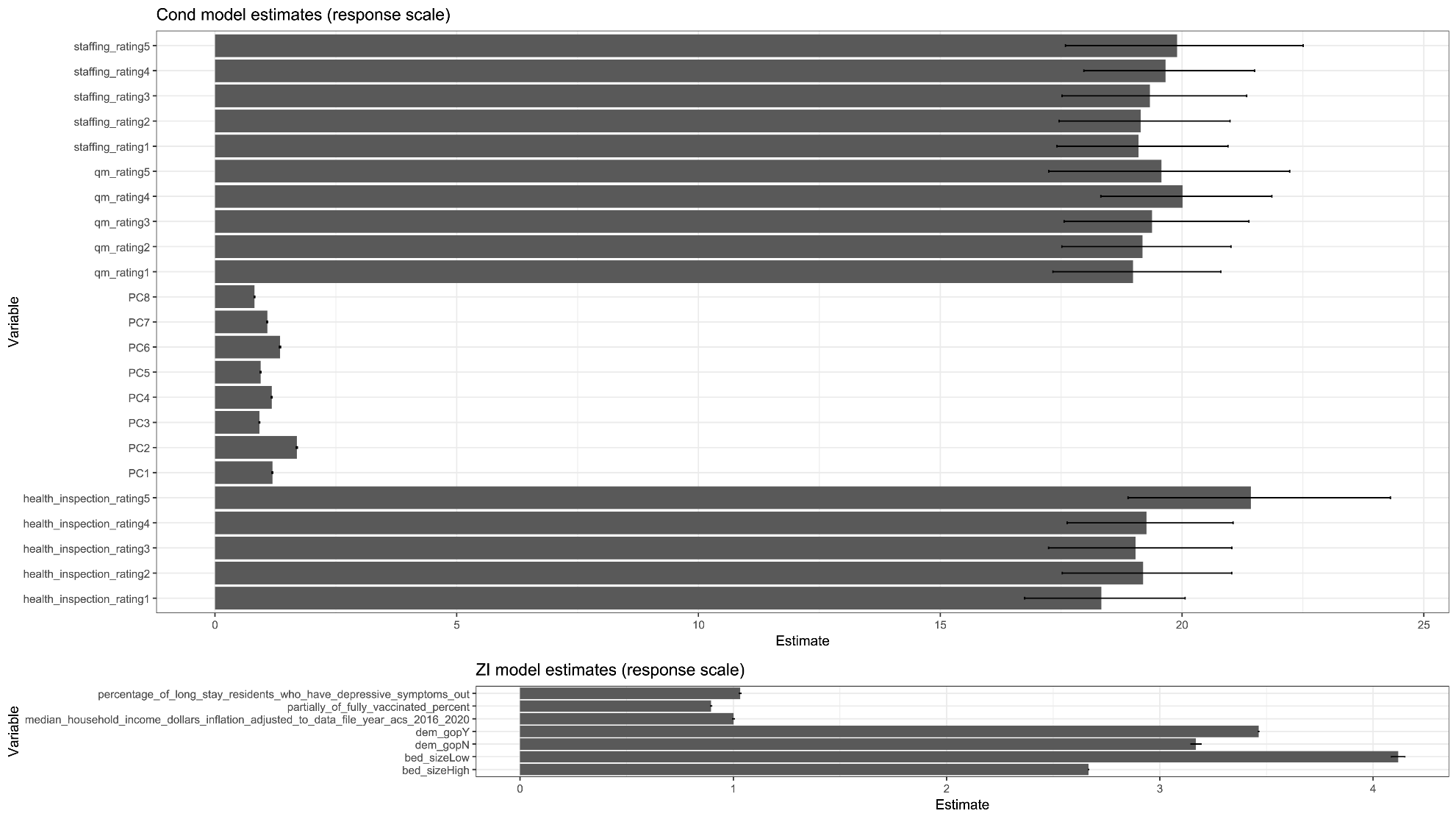


**Figure 20**

Staff infections (model 7 estimates in the response (exponent) scale)


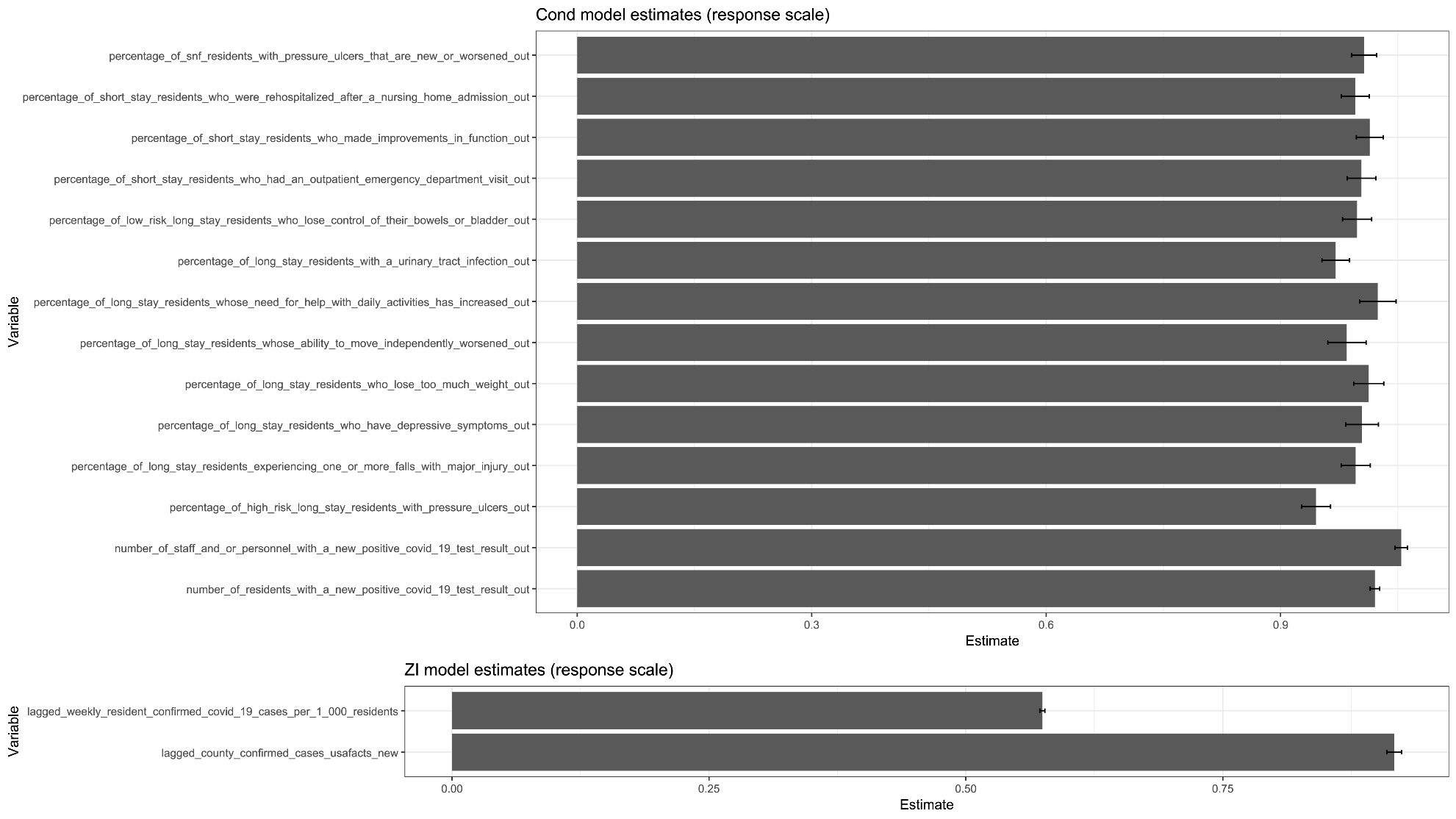


**Figure 21**

Total deaths (model 1 estimates in the response (exponent) scale)


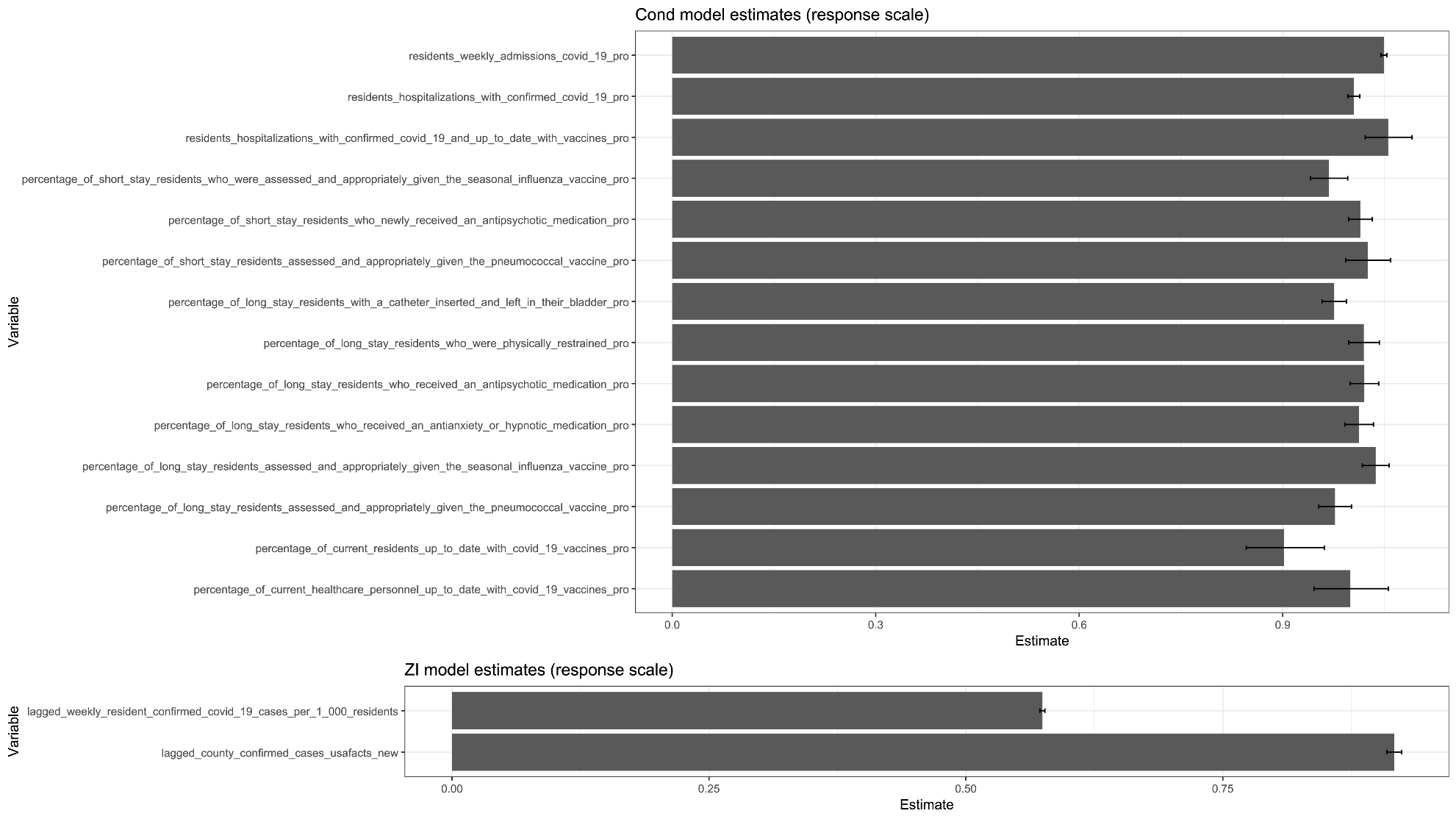


**Figure 22**

Total deaths (model 2 estimates in the response (exponent) scale)


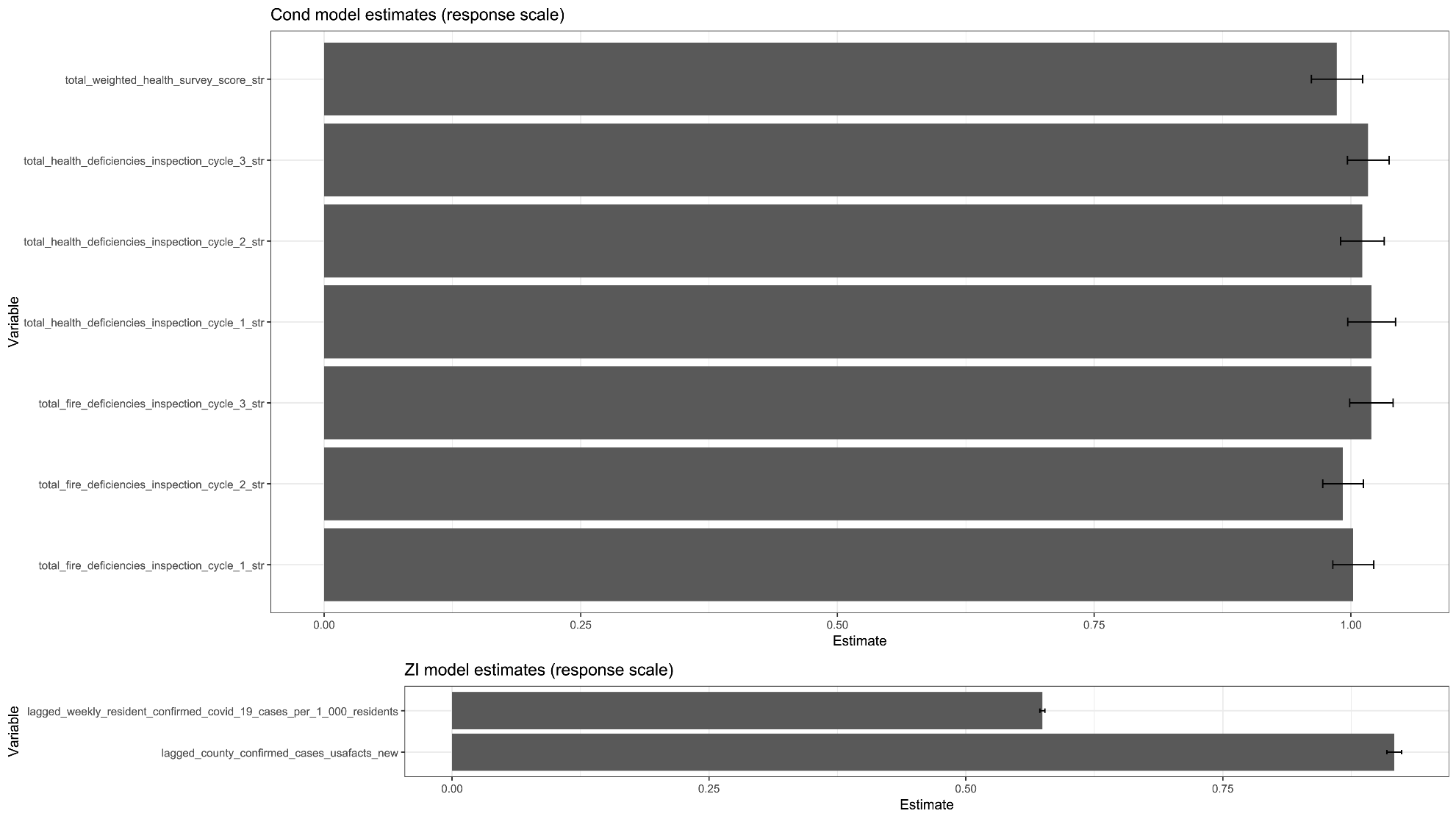


**Figure 23**

Total deaths (model 3 estimates in the response (exponent) scale)


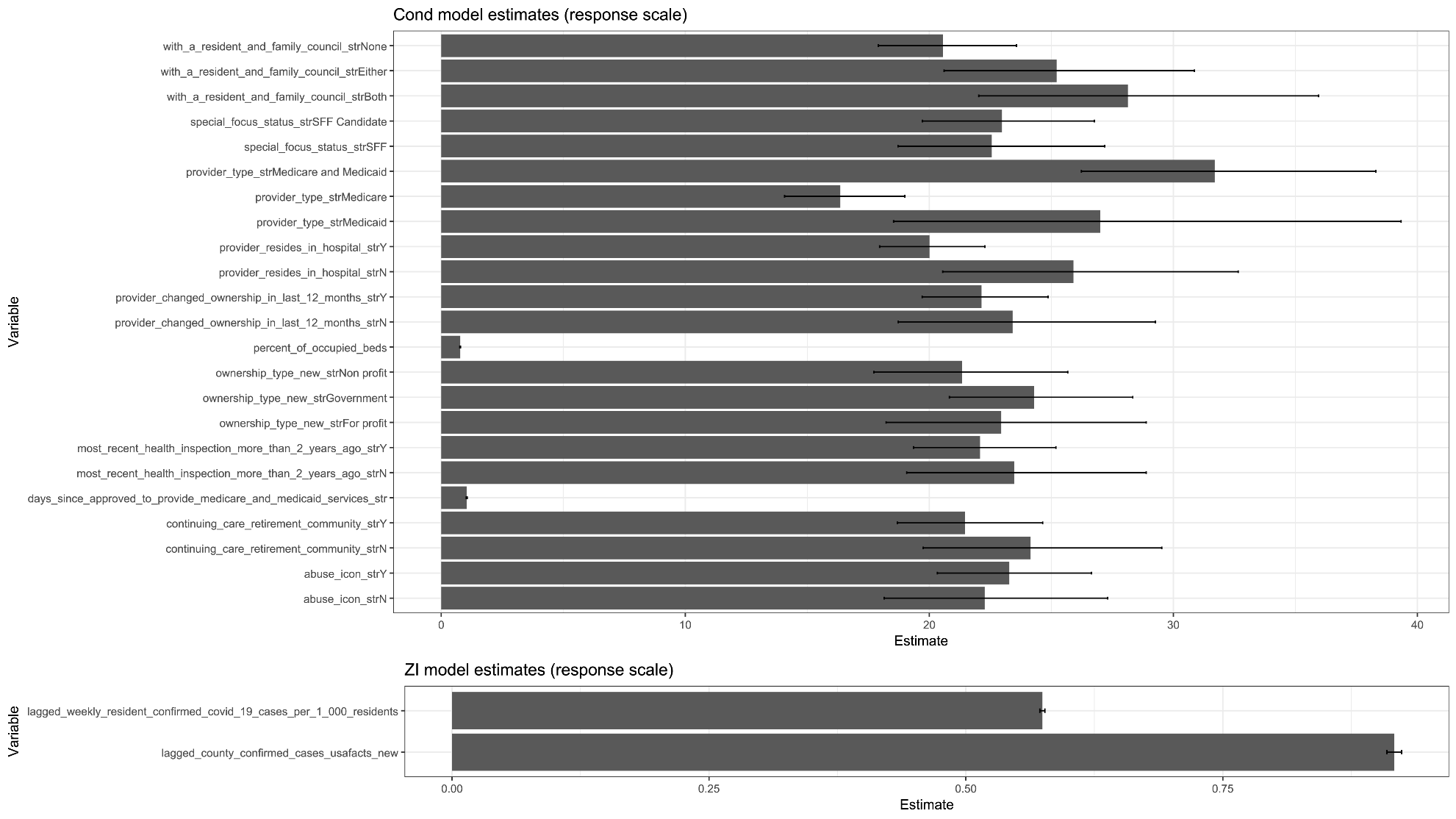


**Figure 24**

Total deaths (model 4 estimates in the response (exponent) scale)


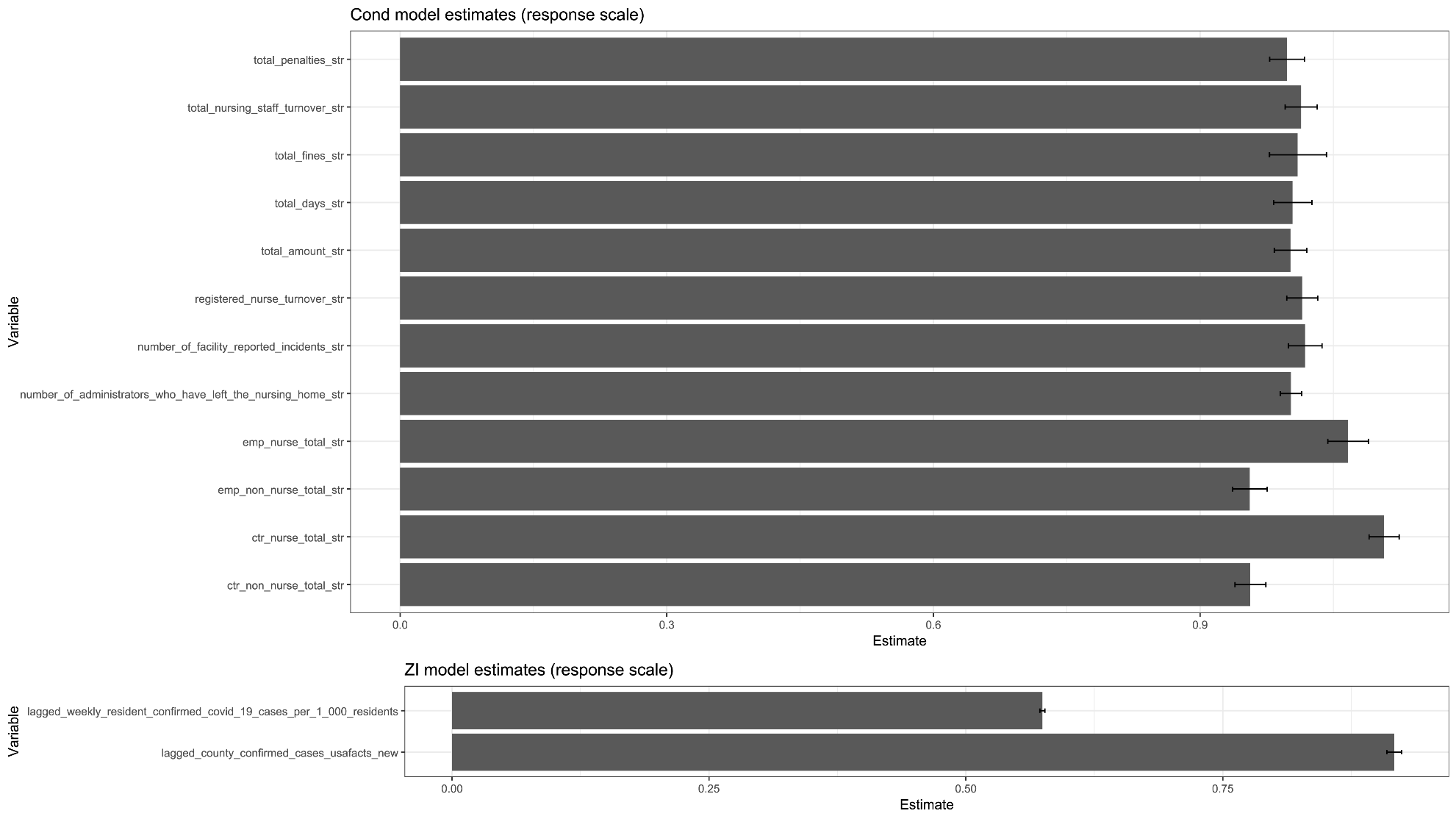


**Figure 25**

Total deaths (model 5 estimates in the response (exponent) scale)


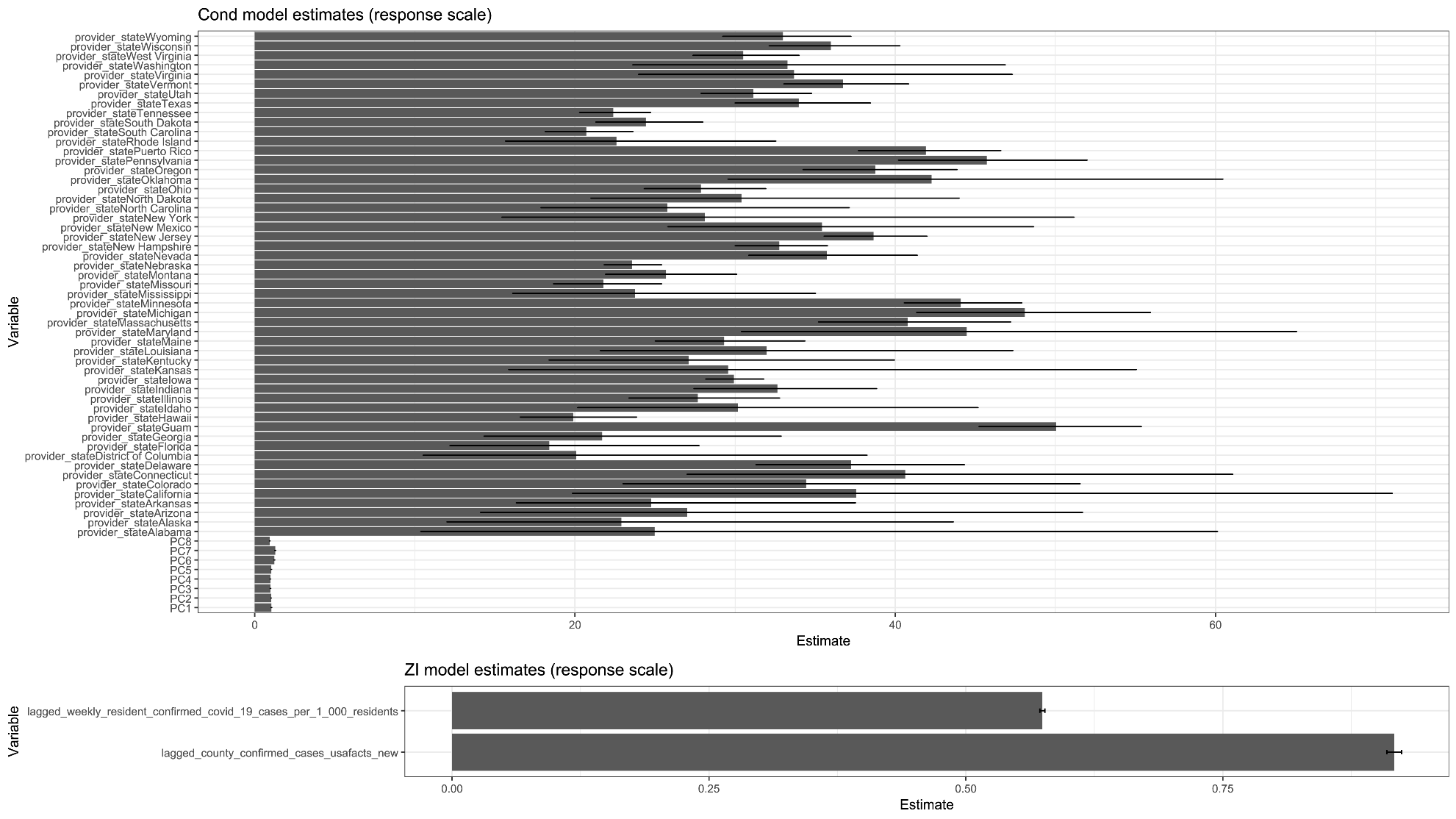


**Figure 26**

Total deaths (model 6 estimates in the response (exponent) scale)


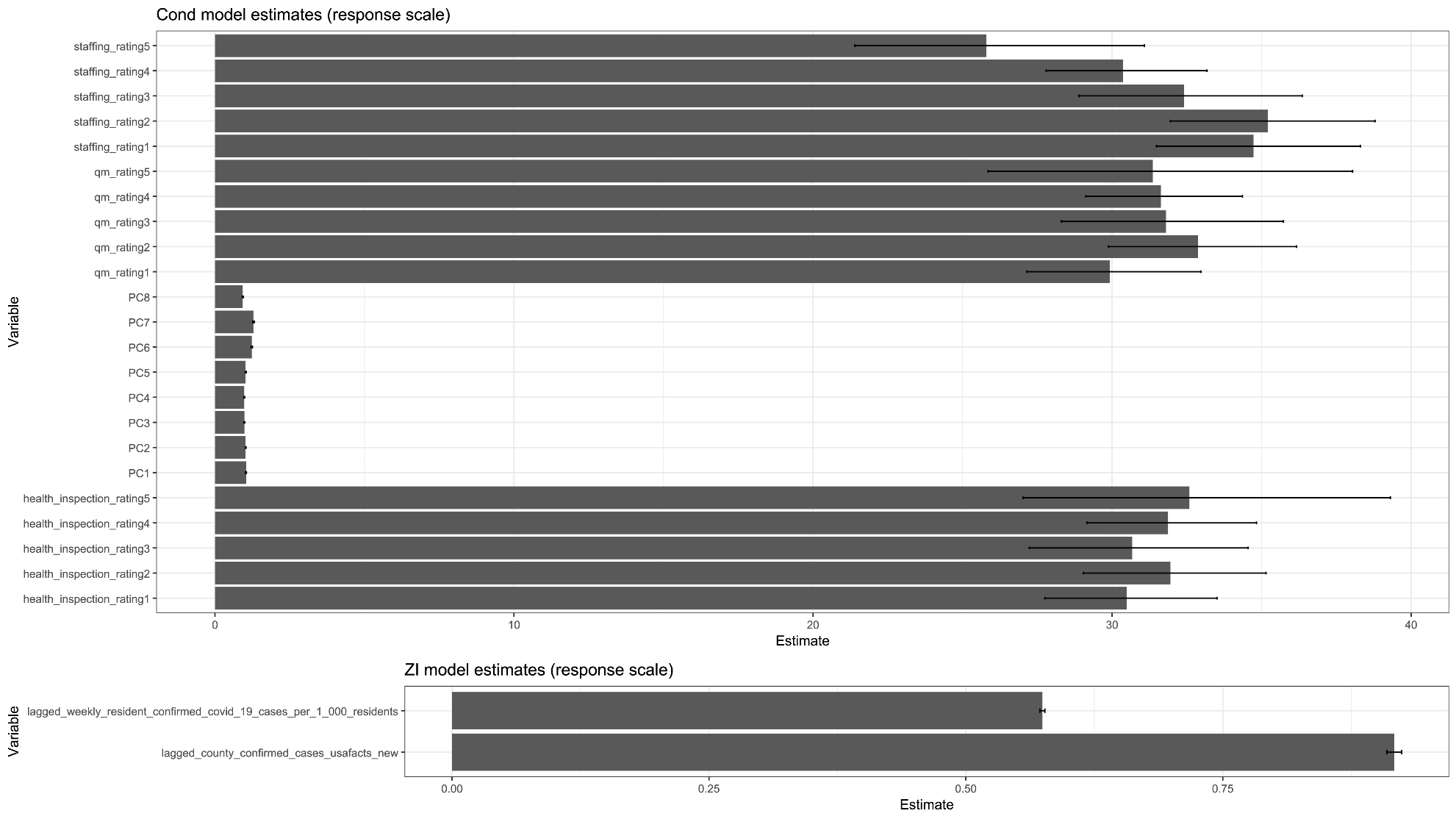


**Figure 27**

Total deaths (model 7 estimates in the response (exponent) scale)
